# Molecular Mechanisms of Biochanin A in AML Cells: Apoptosis Induction and Pathway-Specific Regulation in U937 and THP-1

**DOI:** 10.3390/ijms26115317

**Published:** 2025-05-31

**Authors:** Pei-Shan Wu, Jui-Hung Yen, Pei-Yi Chen, Ming-Jiuan Wu

**Affiliations:** 1 Department of Pharmacy, Chia Nan University of Pharmacy and Science, Tainan 717301, Taiwan; dc7575@gmail.com; 2 Department of Molecular Biology and Human Genetics, Tzu Chi University, Hualien 970374, Taiwan; imyenjh@mail.tcu.edu.tw (J.-H.Y.); pyc571@gmail.com (P.-Y.C.); 3 Institute of Medical Sciences, Tzu Chi University, Hualien 970374, Taiwan; 4 Center of Medical Genetics, Hualien Tzu Chi Hospital, Buddhist Tzu Chi Medical Foundation, Hualien 970473, Taiwan

**Keywords:** Biochanin A, acute myeloid leukemia, apoptosis

## Abstract

Biochanin A, a naturally occurring isoflavone derived from legumes, possesses anti-inflammatory, estrogenic, and anticancer activities. In this study, we investigated the cytotoxic effects and underlying molecular mechanisms of Biochanin A in acute myeloid leukemia (AML) cell lines, U937 and THP-1, using in vitro cytotoxicity assays, RNA sequencing, and bioinformatic analyses. Biochanin A induced dose-dependent apoptosis, as evidenced by caspase-7 activation and PARP1 cleavage. Over-representation analysis (ORA) revealed that differentially expressed genes (DEGs) were significantly enriched in pathways related to inflammatory responses, DNA replication, and cell cycle regulation. Gene set enrichment analysis (GSEA) further confirmed the upregulation of apoptosis- and inflammation-related pathways and the downregulation of MYC targets, cholesterol biosynthesis, and G2/M checkpoint gene sets. RT-qPCR analysis demonstrated that Biochanin A downregulated oncogenes such as *RUNX1*, *BCL2*, and *MYC* while upregulating *CHOP* (*GADD153*), *CDKN1A* (*p21*), and *SQSTM1* (*p62*), contributing to apoptosis and cell cycle arrest across both cell lines. Notably, Biochanin A downregulated *PLK1* and *UHRF1* in THP-1 cells, indicating a disruption of mitotic progression and epigenetic regulation. In contrast, in U937 cells, Biochanin A upregulated *TXNIP* and downregulated *CCND2*, highlighting the involvement of oxidative stress and G1/S cell cycle arrest. These findings support the potential of Biochanin A as a promising therapeutic candidate for AML through both shared and distinct regulatory pathways.

## 1. Introduction

**Figure 1:**
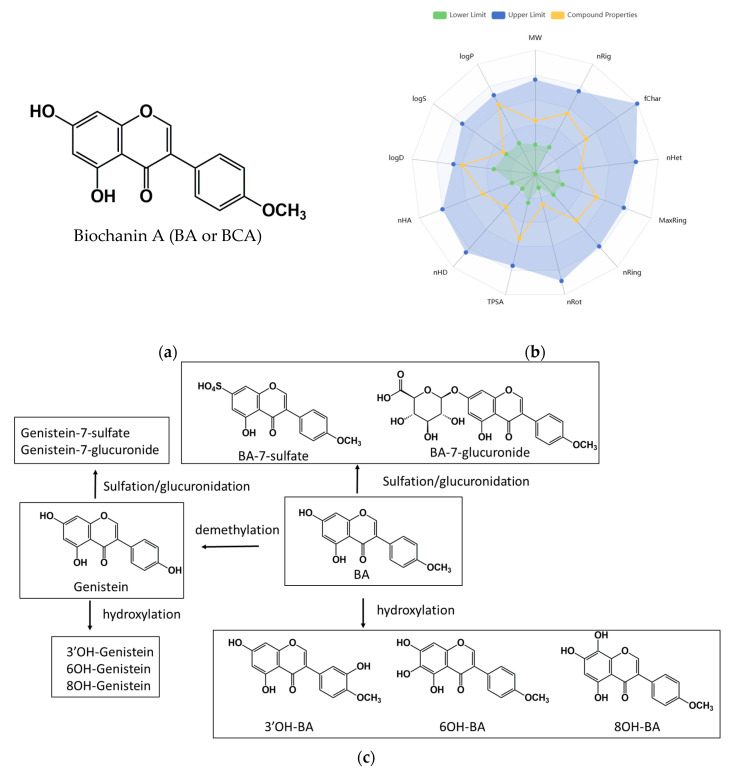
Chemical structure, predicted physicochemical properties, and primary metabolites of Biochanin A. (**a**) Chemical structure of Biochanin A. (**b**) Predicted physicochemical properties of Biochanin A obtained from ADMETlab 3.0 (accessed on 15 January 2025). Abbreviations: MW, molecular weight; nHA, number of hydrogen bond acceptors; nHD, number of hydrogen bond donors; nRot, number of rotatable bonds; nRing, number of rings; MaxRing, number of atoms in the largest ring; nHet, number of heteroatoms; fChar, formal charge; nRig, number of rigid bonds; TPSA, topological polar surface area; logS, water solubility; logP, partition coefficient; logD, distribution coefficient. (**c**) Proposed biotransformation pathway of Biochanin A and its major metabolites.

The phytoestrogen Biochanin A (Figure 1a), also known as 5,7-dihydroxy-4′-methoxyisoflavone, is an isoflavone derivative primarily derived from red clover, soybeans, alfalfa sprouts, peanuts, chickpeas, and other legumes. Biochanin A exhibits a broad spectrum of biological activities, including antioxidant, anti-inflammatory, estrogenic, metabolic regulatory, neuroprotective, and anticancer effects [1]. It has demonstrated cytotoxic or growth-inhibitory activity against various cancer types, including breast, cervical, colorectal, gastric, glioblastoma, liver, lung, melanoma, oral, osteosarcoma, ovarian, pancreatic, pharyngeal, prostate, and umbilical vein cancers [2]. The anticancer mechanisms of Biochanin A involve the inhibition of cell proliferation via the modulation of cyclins and cyclin-dependent kinases, induction of cell cycle arrest, suppression of tumor-promoting signaling pathways, inhibition of angiogenesis and metastasis through downregulation of VEGF and matrix metalloproteinases (MMPs), and activation of apoptosis via modulation of pro- and anti-apoptotic proteins [2,3].

A radar plot illustrating the physicochemical properties of Biochanin A is presented in Figure 1b. These properties were predicted using the ADMETlab 3.0 server (accessed on 15 January 2025) [4]. The ADMET profile suggests that Biochanin A exhibits favorable permeability across Caco-2 and PAMPA membranes, whereas MDCK permeability is poor. Despite its predicted gastrointestinal absorption, the compound demonstrates poor oral bioavailability, particularly at thresholds above 30%. Biochanin A exhibits high plasma protein binding (97.16%) and a very low volume of distribution, indicating limited free drug availability and minimal tissue distribution. It is unlikely to penetrate the blood–brain barrier and is predicted to inhibit key transporters, including OATP1B1, OATP1B3, and BCRP, suggesting a potential for drug–drug interactions [4].

Metabolism predictions indicate that Biochanin A is a strong inhibitor and substrate of multiple cytochrome P450 enzymes, including CYP1A2, CYP2C19, CYP2C9, CYP2D6, CYP3A4, CYP2B6, and CYP2C8, reflecting a high likelihood of metabolic interactions. The human liver microsomal (HLM) stability value (0.388) suggests moderate metabolic stability. The compound exhibits moderate plasma clearance (CL_plasma_ = 5.446 mL/min/kg), while its predicted half-life (T_1/2_ = 0.858 h) is classified as ultra-short, indicating the need for frequent dosing [4].

Toxicity predictions reveal a high risk of drug-induced liver injury (DILI), genotoxicity, carcinogenicity, respiratory toxicity, and eye irritation. Moderate risks were observed for AMES mutagenicity, skin sensitization, human hepatotoxicity, and Hek293 cytotoxicity. Conversely, Biochanin A shows low predicted risk for hERG channel inhibition (cardiotoxicity), nephrotoxicity, ototoxicity, hematotoxicity, and immunotoxicity [4].

The physicochemical analysis indicates that Biochanin A exhibits favorable drug-likeness properties, satisfying the criteria defined by Lipinski, Ghose, Veber, Egan, and Muegge, as evaluated using the SwissADME platform (http://www.swissadme.ch/, accessed on 15 January 2025) [5]. In humans, Biochanin A undergoes extensive metabolism, yielding multiple bioactive metabolites. As shown in Figure 1c, Biochanin A is demethylated to produce genistein, a structurally related isoflavone. This transformation is catalyzed by intestinal microbiota or hepatic microsomal enzymes. Both Biochanin A and genistein are further metabolized via hydroxylation at various positions (3′-, 6-, and 8-hydroxy derivatives) through the action of hepatic cytochrome P450 enzymes. In addition, Phase II conjugation reactions lead to the formation of glucuronide and sulfate conjugates, mediated by glucuronosyltransferases and sulfotransferases, respectively [6,7].

As a phytoestrogen, Biochanin A is capable of binding to estrogen receptors (ERs). Unlike other isoflavones such as genistein and daidzein, which may antagonize chemotherapeutic agents like cisplatin or tamoxifen in hormone-sensitive cancers [8,9], Biochanin A exhibits significantly lower affinity for ERα and ERβ—over 10,000-fold lower than that of estradiol [9,10]. This weak binding suggests a reduced potential for endocrine disruption and a potentially safer profile for use in hormone-sensitive conditions.

Acute myeloid leukemia (AML) is a heterogenous disease that arises from uncontrolled proliferation of clonal hematopoietic cells [11]. AML is a complex disease that encompasses several subtypes, each presenting with a diverse array of genetic abnormalities and variable prognoses [12]. Next-generation sequencing (NGS) identifies recurrent molecular abnormalities in 90% of individuals with AML. The most frequent mutations are *FLT3*, *NPM1*, *DNMT3A*, *IDH1*, *IDH2*, *TET2*, *RUNX1*, *TP53*, *NRAS*, *CEBPA*, and *WT1*, which can be observed as single mutations or, more often, concurrently with other mutations. AML mutations can be prognostic and/or predictive. In some cases, they are actionable targets [13]. In addition to traditional 7 + 3 anthracycline-cytarabine regimens, several targeted agents, such as BCL2, FLT3, IDH, DNMT, and menin inhibitors, have been approved by the FDA since 2017 [13].

The U937 (also known as U-937) is a human pro-monocytic myeloid leukemia cell line derived from an adult white male [14]. In contrast, the THP-1 cell line is a human monocytic leukemia cell line derived from the peripheral blood of a 1-year-old boy [15]. They are among the most commonly used AML cell lines. Cytarabine (Ara-C) has been reported to induce apoptosis in both U937 and THP-1 cells through a similar mechanism [16]. However, significant differences emerge during macrophage differentiation: THP-1-derived macrophages exhibit heightened responsiveness to pro-inflammatory (M1) stimuli, whereas U937-derived macrophages display a bias toward the M2/alternative phenotype upon treatment with the protein kinase C (PKC) activator phorbol-12-myristate-13-acetate (TPA) [17]. These findings suggest the presence of intrinsic differences in cellular responses to drug treatments. In fact, U937 harbors six cancer driver cancer genes, *JAK3*, *RNF43*, *TP53*, *PTPN11*, *WT1*, and *PTEN*, while THP-1 contains three: *NRAS*, *TP53*, and *SDHA* [18].

In this study, we investigated the effects and molecular mechanisms of Biochanin A on AML cells using an integrated in vitro and in silico approach. The cytotoxic effects of Biochanin A were first evaluated in U937 and THP-1 cell lines. RNA sequencing (RNAseq) was subsequently performed to identify differentially expressed genes (DEGs) in these cell lines following Biochanin A treatment. Over-representation analysis (ORA) was conducted to determine the enrichment of DEGs in specific Gene Ontology (GO) terms and Kyoto Encyclopedia of Genes and Genomes (KEGG) pathways [19,20,21]. Gene set enrichment analysis (GSEA) was then employed to verify whether Biochanin A treatment activated/inhibited the functional pathways [22]. Finally, the expression of AML-related genes was validated by RT-qPCR.

## 2. Results

### 2.1. Effects of Biochanin A on the Viabilities of U937 and THP-1 Cells

**Figure 2:**
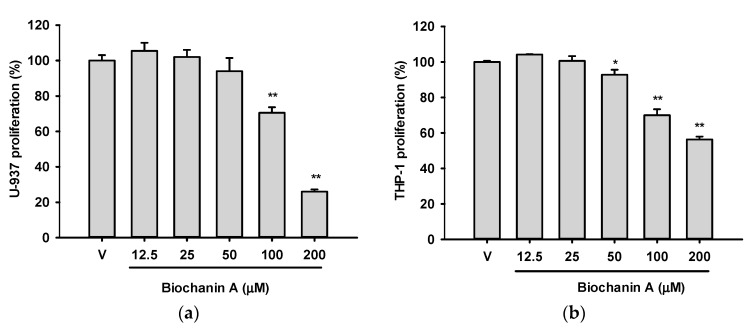

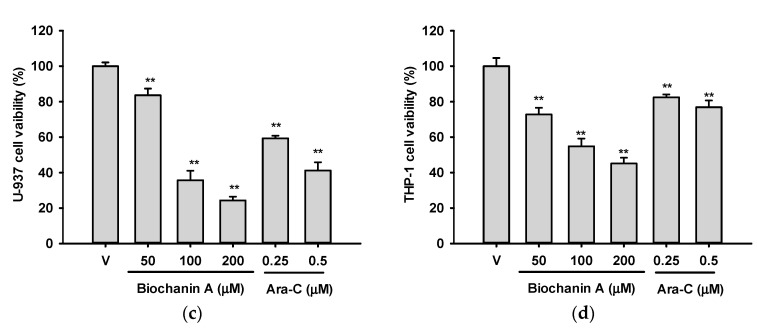
The effects of Biochanin A on cell proliferation and viability in U937 and THP-1 cells. (**a**,**b**) U937 and THP-1 cells were treated with the vehicle control (0.1% DMSO) or Biochanin A (12.5–100 μM) for 24 h, and cell proliferation was assessed using the MTT assay. (**c**,**d**) Cell viability was determined by the trypan blue exclusion assay following 24 h treatment with Biochanin A. All experiments were performed in triplicate, and data are presented as the mean ± SD of three independent experiments. * *p* < 0.05 and ** *p* < 0.01 indicate statistically significant differences compared with vehicle-treated controls.

Cell proliferation was initially evaluated using the MTT assay following treatment of U937 and THP-1 cells with increasing concentrations of Biochanin A (12.5–200 μM) for 24 h. As shown in Figure 2a,b, Biochanin A significantly inhibited cell viability at concentrations ≥100 μM in U937 cells and ≥50 μM in THP-1 cells.

To further assess cytotoxicity, trypan blue exclusion analysis combined with light microscopy was performed after 24 h treatment with Biochanin A (50–200 μM). Cytarabine (Ara-C; 0.25 and 0.5 μM) was used as a positive control (Figure 2c,d). The trypan blue assay revealed stronger cytotoxic effects than those detected by the MTT assay. This discrepancy may be attributed to the reductive properties of Biochanin A, which can artificially reduce MTT to formazan, leading to an overestimation of cell viability independent of mitochondrial function [23]. Notably, Biochanin A at concentrations ≥100 μM exhibited significantly higher cytotoxicity in U937 cells compared to THP-1 cells (*p* < 0.05). Similarly, Ara-C also showed greater cytotoxicity in U937 cells than in THP-1 cells.

### 2.2. Effects of Biochanin A on Cell Proliferation- and Apoptosis-Related Protein Expression in U937 and THP-1 Cells

**Figure 3:**
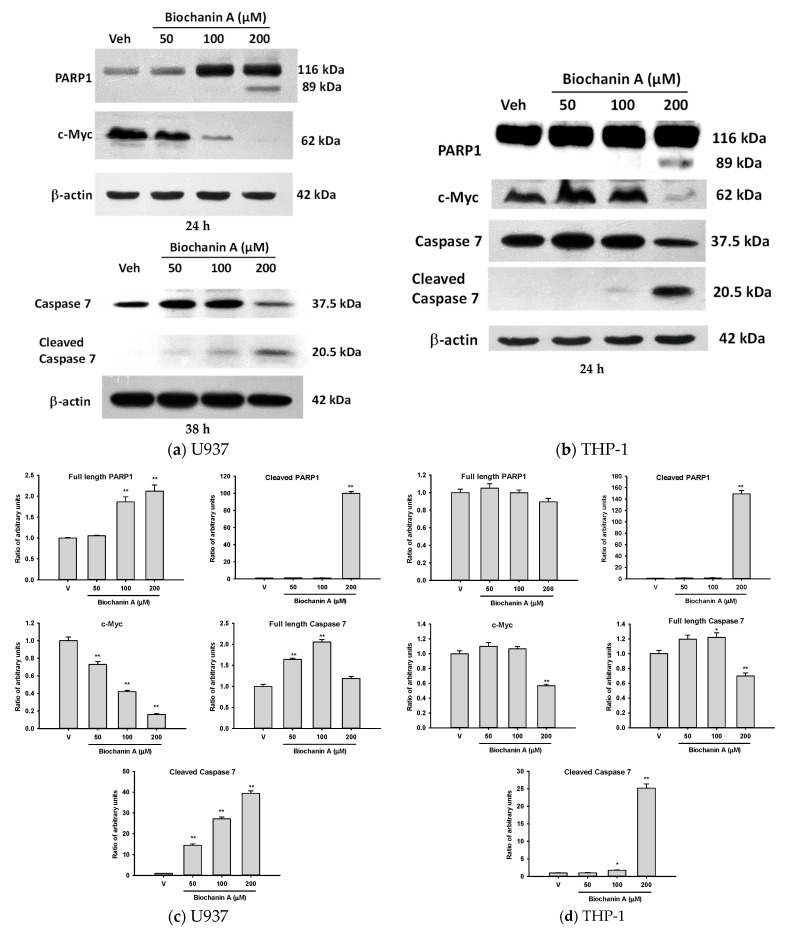
Biochanin A induces PARP-1 cleavage, activates caspase-7, and suppresses c-MYC protein expression in U937 and THP-1 cells. (**a**,**b**) The Western blot analysis of PARP-1, cleaved caspase-7, and c-MYC in U937 and THP-1 cells following treatment with Biochanin A for the indicated time periods. (**c**,**d**) The densitometric quantification of protein band intensities using ImageJ for U937 and THP-1 cells, respectively. Total cell lysates were collected and analyzed as described in the Materials and Methods section. Data are presented as the mean ± SD from three independent experiments. * *p* < 0.05 and ** *p* < 0.01 indicate statistically significant differences compared with vehicle-treated controls.

Based on the results of the MTT and trypan blue exclusion assays, Biochanin A was found to inhibit cell proliferation and induce cell death in both U937 and THP-1 cells. Poly (ADP-ribose) polymerase-1 (PARP-1) is a critical mediator of the cellular response to DNA damage, playing a key role in DNA repair and the maintenance of genomic stability [24]. During apoptosis, PARP-1 is cleaved by activated caspase-3 and caspase-7, resulting in the inactivation of its enzymatic activity [25]. As shown in Figure 3a,c, treatment with Biochanin A (100 and 200 μM) for 24 h increased the expression of full-length PARP-1 (~116 kDa) in U937 cells, with a notable accumulation of the cleaved form (~89 kDa) at 200 μM. These findings indicate that Biochanin A induces a DNA damage response and activates apoptotic signaling in a dose-dependent manner.

Interestingly, caspase-7 upregulation and cleavage were not observed in U937 cells until 38 h following treatment with Biochanin A (50–200 μM), indicating a delayed activation. This delay suggests that caspase-7 protein levels may have been below the detection threshold at 24 h. In contrast, as shown in Figure 3b,d, Biochanin A (100–200 μM) induced the expression and cleavage of caspase-7 in THP-1 cells after 24 h of treatment. These findings highlight cell-type-specific differences in the kinetics of apoptotic signaling in response to Biochanin A.

MYC is a key regulator of ribosome biogenesis and plays a central role in promoting cell growth and proliferation [26,27]. Treatment with Biochanin A (50–200 μM) resulted in a dose-dependent reduction of c-MYC protein expression in U937 cells (Figure 3a,c). Notably, PARP-1 cleavage and a decrease in c-MYC protein levels were only observed at the highest concentration tested (200 μM) in THP-1 cells, suggesting a threshold effect for apoptosis induction (Figure 3b,d).

### 2.3. Transcriptome Analysis for Biochanin A-Treated U937 Cells: Over-Representation Analysis (ORA) and Gene Set Enrichment Analysis (GSEA)

Transcriptome analysis provides a comprehensive means of evaluating changes in RNA expression under stress conditions and serves as a valuable tool for guiding early-phase drug discovery decisions [28]. In our previous studies, we have routinely selected a treatment dose and time point that results in approximately 50% or slightly greater cell death for transcriptomic profiling [29,30,31]. This strategy ensures that the observed gene expression changes are biologically relevant to cytotoxic effects while preserving enough viable cells for meaningful analysis.

**Figure 4:**
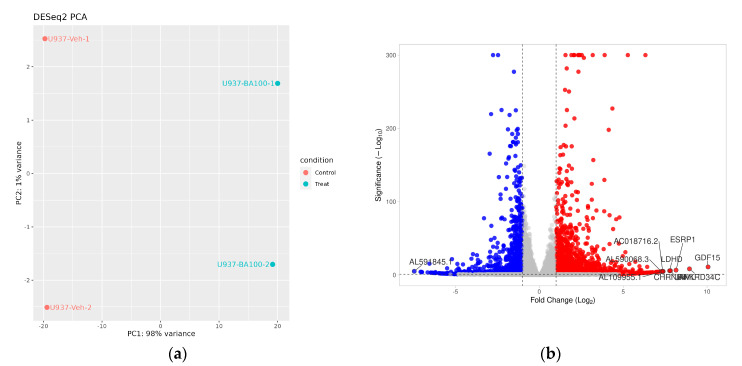
Biochanin A (100 μM) induces transcriptomic alterations in U937 cells. (**a**) Principal component analysis (PCA) plot showing distinct clustering between vehicle-treated (Veh) and Biochanin A-treated (BA100) samples, indicating transcriptomic variation. (**b**) Volcano plot illustrating differentially expressed genes (DEGs) in U937 cells following treatment with 100 μM Biochanin A. Vertical lines represent log_2_ fold change thresholds (−1 and 1), and the horizontal line indicates the significance threshold (*p* = 0.05). Red dot indicates upregulated gene, while blue dot indicates downregulated gene. The volcano plot was generated using the VolcaNoseR tool (https://huygens.science.uva.nl/VolcaNoseR/, accessed on 15 December 2024) [32].

Following this approach, next-generation sequencing (NGS) was performed to investigate transcriptomic alterations induced by Biochanin A (100 μM) in U937 cells after 24 h of treatment. Principal component analysis (PCA) demonstrated a clear separation between control and Biochanin A-treated samples, with principal component 1 (PC1) accounting for 98% of the total variance (Figure 4a). The differential expression analysis identified a total of 3074 differentially expressed genes (DEGs), comprising 1844 upregulated and 1230 downregulated genes (log_2_|fold change| > 1, *p* < 0.05).

The top ten differentially expressed genes (DEGs) with the largest fold changes were annotated and are shown in Figure 4b. Among the upregulated protein-coding genes were *GDF15* (a marker of inflammation), *LDHD* (involved in mitochondrial metabolism), *JAML* and *ANKRD34C* (associated with cell cycle regulation), *ESRP1* (a regulator of mRNA splicing), and *CHRNB4* (a subunit of the acetylcholine receptor). In addition, several non-coding RNAs, including *AL109955.1*, *AC018716.2*, and *AL590068.3* (upregulated) as well as *AL591845.1* (downregulated), showed significant expression changes following Biochanin A treatment. These findings underscore the extensive transcriptomic remodeling induced by Biochanin A and its regulatory influence on both protein-coding and non-coding genes involved in key cellular functions.

**Figure 5:**
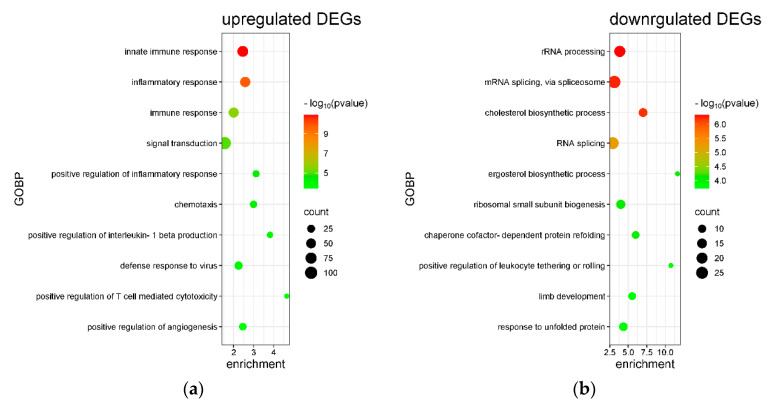

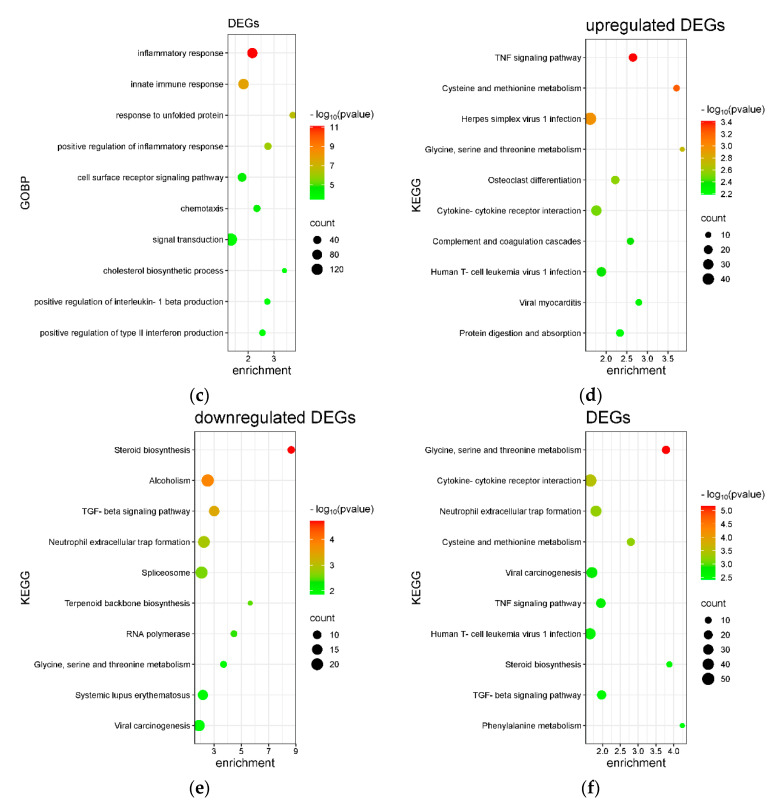
Enrichment analysis of Gene Ontology Biological Processes (GOBPs) and KEGG pathways for Biochanin A (100 μM)-induced differentially expressed genes (DEGs) in U937 cells. (**a**) Top 10 GOBP terms enriched among upregulated DEGs. (**b**) Top 10 GOBP terms enriched among downregulated DEGs. (**c**) Top 10 GOBP terms enriched among all DEGs. (**d**) Top 10 KEGG pathways enriched among upregulated DEGs. (**e**) Top 10 KEGG pathways enriched among downregulated DEGs. (**f**) Top 10 KEGG pathways enriched among all DEGs. Pathways are ranked by statistical significance (*p* value). Bubble size indicates the number of significant genes in each term, while bubble color represents −log_10_ (*p* value). Plots were generated using SRplot (https://www.bioinformatics.com.cn/en?keywords=bubble, accessed on 16 December 2024) [33].

An enrichment analysis of upregulated and downregulated DEGs was performed using the Database for Annotation, Visualization, and Integrated Discovery (DAVID). The results of Gene Ontology Biological Process (GOBP) enrichment are presented in Figure 5a–c. Among the upregulated DEGs as well as the total set of DEGs, the most significantly enriched biological processes were related to innate immune responses and inflammatory signaling. In contrast, downregulated DEGs were significantly enriched in pathways associated with RNA processing and cholesterol biosynthesis. These findings suggest that treatment with 100 μM Biochanin A modulates immune activation while suppressing metabolic and transcriptional regulatory pathways in U937 cells.

**Figure 6:**
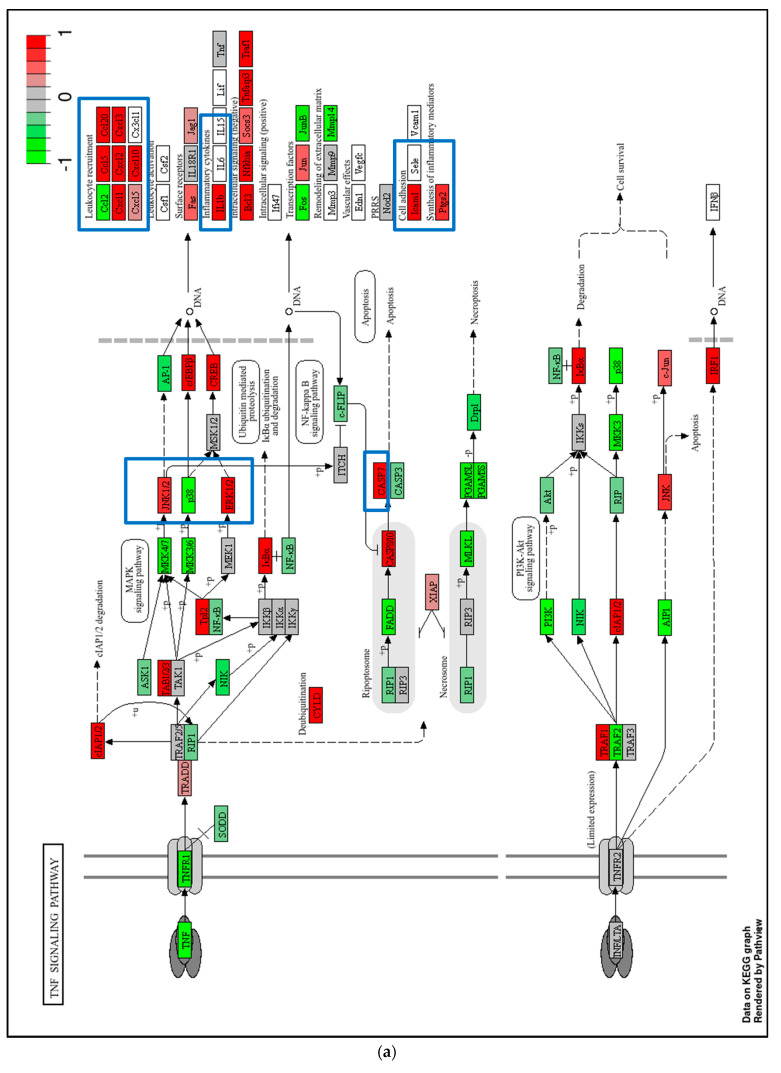

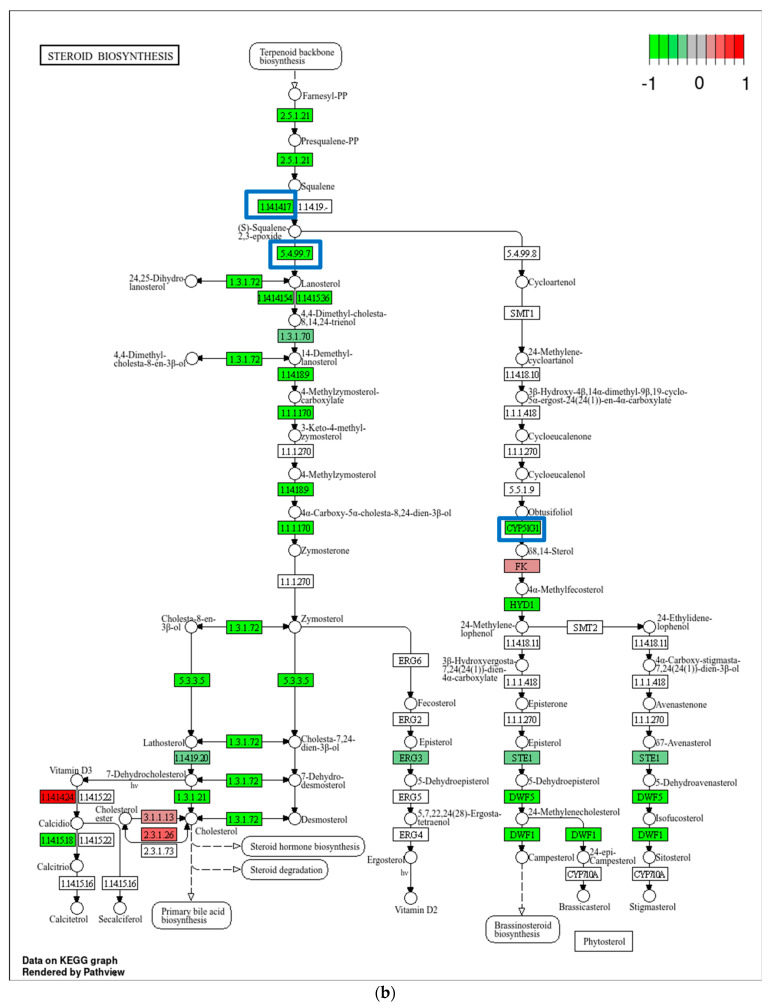
Representative KEGG pathway maps enriched with differentially expressed genes (DEGs) following Biochanin A (100 μM) treatment in U937 cells. (**a**) The TNF signaling pathway enriched with upregulated DEGs. (**b**) The steroid biosynthesis pathway enriched with downregulated DEGs. In both panels, red indicates upregulated genes and green indicates downregulated genes. Genes discussed in the main text are highlighted with a blue box.

KEGG pathway enrichment analysis revealed that the upregulated DEGs were primarily associated with the tumor necrosis factor (TNF) signaling pathway and amino acid metabolism (Figure 5d), whereas the downregulated DEGs were mainly enriched in steroid biosynthesis and RNA polymerase-related pathways (Figure 5e). The specific genes involved in the KEGG TNF signaling and steroid biosynthesis pathways are illustrated in Figure 6.

As shown in Figure 6a, KEGG mapping of the TNF signaling pathway indicates that genes associated with the MAPK cascade, including members of the JNK and ERK subfamilies, were upregulated in U937 cells treated with 100 μM Biochanin A. This upregulation is predicted to enhance the expression of downstream genes involved in leukocyte recruitment, inflammation, and cell adhesion. Additionally, *CASP7* induction was observed, consistent with the Western blot results presented in Figure 3. These findings suggest that Biochanin A-induced apoptosis in U937 cells may be mediated, at least in part, through the activation of MAPK signaling and pro-inflammatory pathways.

As shown in Figure 6b, Biochanin A treatment led to the coordinated downregulation of *SQLE* (squalene monooxygenase; EC 1.14.14.17), *LSS* (lanosterol synthase; EC 5.4.99.7), and *CYP51A1* (sterol 14α-demethylase, formerly CYP51G1), which are key enzymes involved in the early and intermediate stages of cholesterol biosynthesis. This suppression suggests a substantial inhibition of sterol production in U937 cells. The disruption of cholesterol biosynthesis may impair membrane integrity, lipid-mediated signaling, and overall cellular homeostasis, thereby contributing to the cytotoxic and pro-apoptotic effects observed following Biochanin A treatment.

Gene set enrichment analysis (GSEA) was performed to validate the differential expression results. The top ten Hallmark gene sets enriched with upregulated and downregulated genes are presented in Supplemental Figure S1. Upregulated genes were predominantly associated with Hallmark pathways related to cytokine response, inflammation, apoptosis, and xenobiotic metabolism, whereas downregulated genes were significantly enriched in the Wnt/β-catenin signaling pathway, cholesterol biosynthesis, and late estrogen response. The Wnt/β-catenin signaling pathway plays a critical role in embryonic development, stem cell maintenance, tissue homeostasis, and tumorigenesis, primarily by regulating target genes such as *c-MYC*, and *CCND1* (cyclin D1) [34].

**Figure 7:**
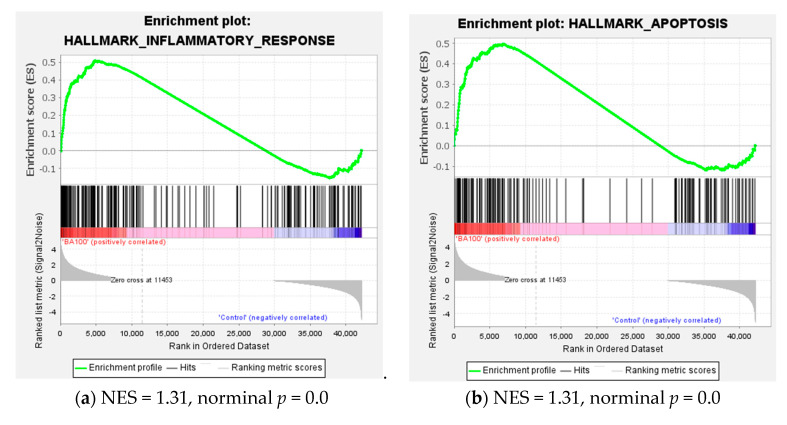

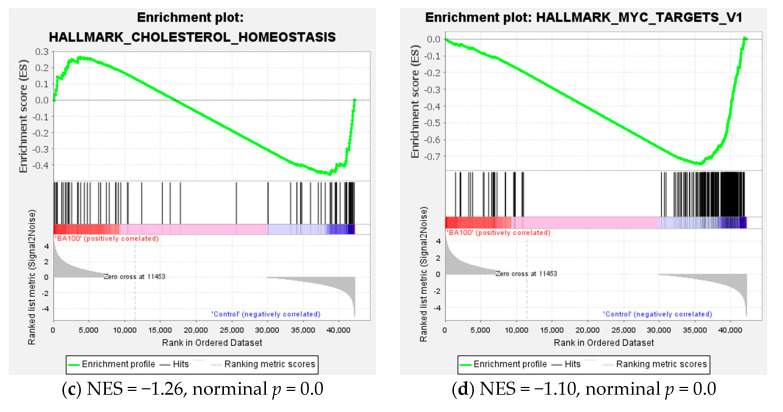
Gene set enrichment analysis (GSEA) plots of Hallmark pathways affected by Biochanin A (100 μM) treatment in U937 cells. (**a**,**b**) Gene sets significantly enriched among upregulated genes. (**c**,**d**) Gene sets significantly enriched among downregulated genes.

GSEA plots of Hallmark pathways related to inflammatory response, apoptosis, cholesterol homeostasis, and MYC targets are presented in Figure 7. In the cholesterol homeostasis pathway (Figure 7c), Biochanin A treatment led to a significant downregulation of several key genes beyond *SQLE* and *LSS*, including *HMGCR* (3-hydroxy-3-methylglutaryl-CoA reductase), *HMGCS1* (3-hydroxy-3-methylglutaryl-CoA synthase 1), and *SREBF2* (sterol regulatory element-binding transcription factor 2). These findings indicate that Biochanin A markedly suppresses the transcriptional program governing cholesterol biosynthesis and homeostasis.

### 2.4. Transcriptome Analysis for Biochanin A-Treated THP-1 Cells: ORA and GSEA

**Figure 8:**
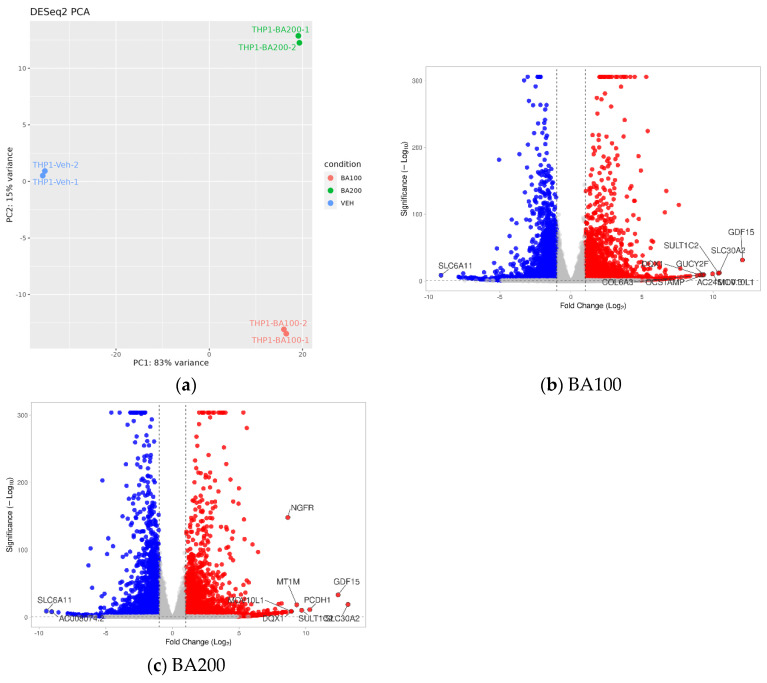
Transcriptomic alterations in THP-1 cells following treatment with Biochanin A (100 and 200 μM). (**a**) The principal component analysis (PCA) plot showing transcriptome profiles of vehicle-treated cells (Veh) and cells treated with 100 μM (BA100) and 200 μM (BA200) Biochanin A. (**b**,**c**) Volcano plots showing differentially expressed genes (DEGs) in THP-1 cells treated with 100 μM (**b**) and 200 μM (**c**) Biochanin A. Vertical lines represent log_2_ fold change thresholds (−1 and 1), and the horizontal line indicates the significance threshold (*p* = 0.05). Red dot indicates upregulated gene, while blue dot indicates downregulated gene. Volcano plots were generated using VolcaNoseR (https://huygens.science.uva.nl/VolcaNoseR/, accessed on 15 December 2024) [32].

The cell viability analysis showed that treatment with 100 μM and 200 μM Biochanin A resulted in approximately 45% and 55% cell death, respectively, after 24 h. Based on these findings, transcriptome analysis was performed at both doses following 24 h treatment. A principal component analysis (PCA) was conducted to evaluate differences in gene expression profiles among treatment groups in THP-1 cells. The analysis revealed clear separation between the vehicle-treated (Veh), 100 μM Biochanin A (BA100), and 200 μM Biochanin A (BA200) groups, with principal component 1 (PC1) accounting for 83% of the total variation (Figure 8a).

Biochanin A treatment resulted in 5174 and 5217 differentially expressed genes (DEGs) for 100 μM and 200 μM doses, respectively. Specifically, 100 μM Biochanin A led to 2790 upregulated and 2384 downregulated DEGs, while 200 μM Biochanin A caused 2795 upregulated and 2422 downregulated DEGs (Figure 8b,c). Notably, 1889 upregulated and 1299 downregulated DEGs overlapped between the two doses. The top upregulated DEGs shared by both 100 μM and 200 μM Biochanin A treatments included protein-coding genes such as *GDF15* (inflammation marker), *SLC30A2* (lysosomal-mediated cell death), *SULT1C2* (sulfotransferase activity), and RNA helicases *MOV10L1* and *DQX1*, along with the non-coding RNA *AC245100.3.* In comparison, 561 upregulated and 394 downregulated DEGs were shared between THP-1 and U937 cells (Supplemental Figure S2).

**Figure 9:**
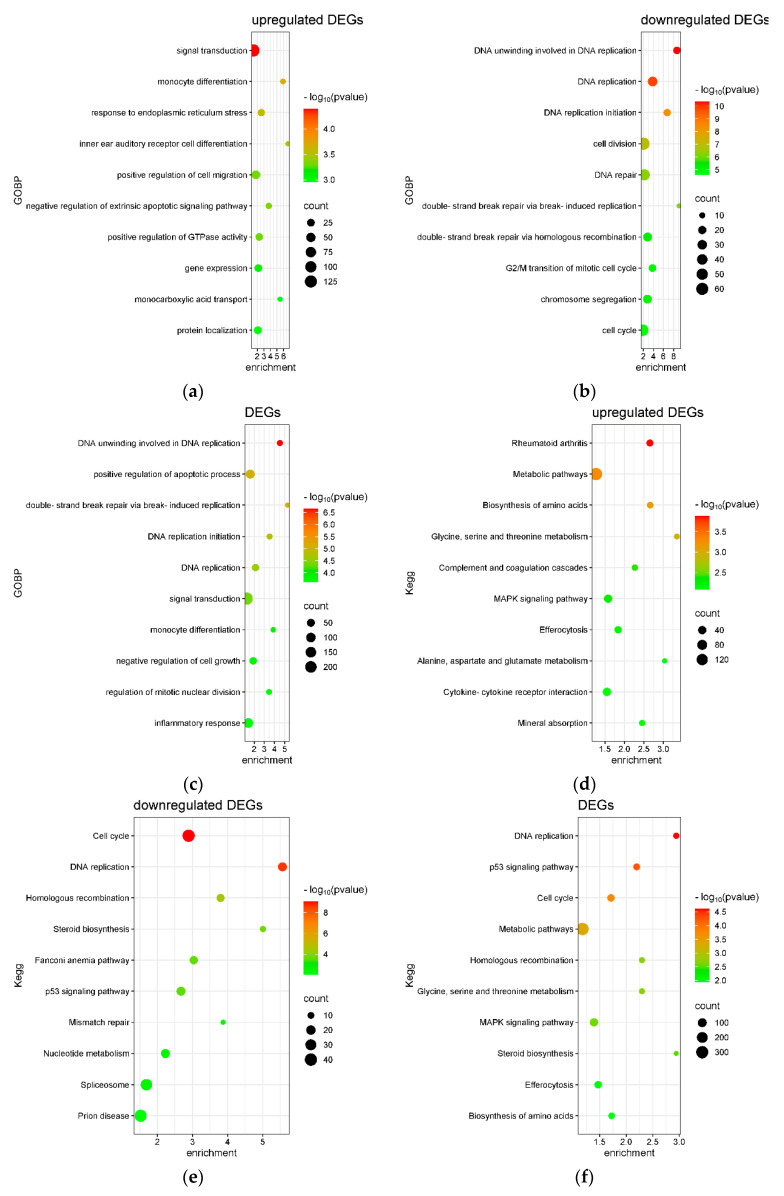
The enrichment analysis of Gene Ontology Biological Processes (GOBP) and KEGG pathways for differentially expressed genes (DEGs) induced by Biochanin A (100 μM) in THP-1 cells. (**a**) Top 10 GOBP terms enriched among upregulated DEGs. (**b**) Top 10 GOBP terms enriched among downregulated DEGs. (**c**) Top 10 GOBP terms enriched among all DEGs. (**d**) Top 10 KEGG pathways enriched among upregulated DEGs. (**e**) Top 10 KEGG pathways enriched among downregulated DEGs. (**f**) Top 10 KEGG pathways enriched among all DEGs. Pathways are ranked by statistical significance (*p* value). The bubble size indicates the number of DEGs associated with each term, and the bubble color reflects −log_10_ (*p* value). Plots were generated using SRplot (https://www.bioinformatics.com.cn/en?keywords=bubble, accessed on 16 December 2024) [33].

**Figure 10:**
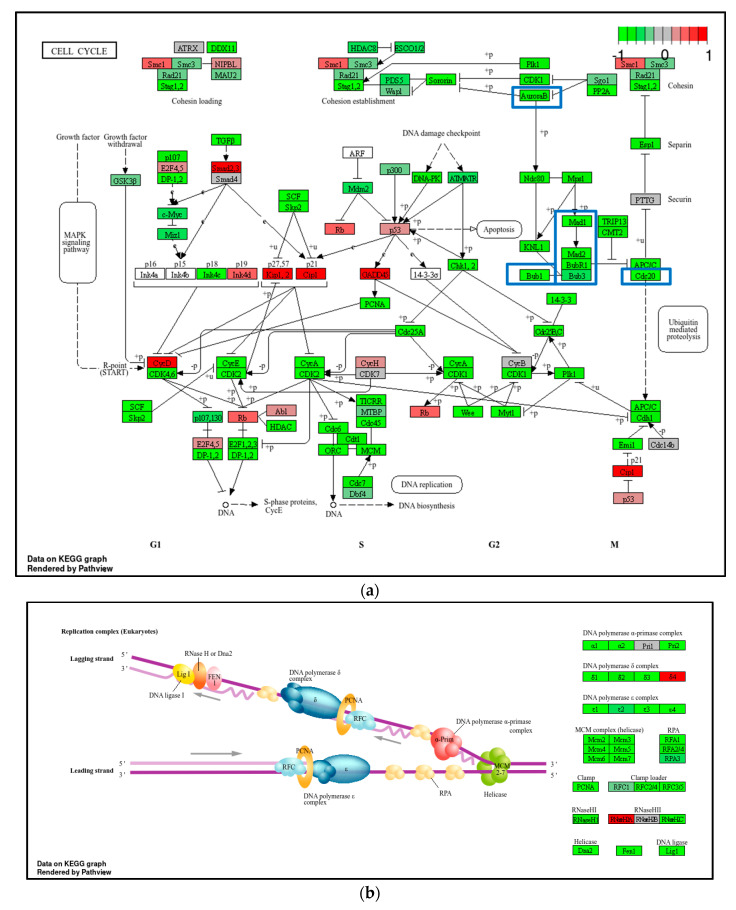
KEGG pathway maps enriched with downregulated differentially expressed genes (DEGs) in THP-1 cells following treatment with Biochanin A (100 μM). (**a**) The cell cycle pathway. (**b**) The eukaryotic DNA replication pathway. Red indicates upregulated genes, and green indicates downregulated genes. Genes discussed in the main text are highlighted with a blue box.

Figure 9a–c present the Gene Ontology Biological Process (GOBP) terms enriched among differentially expressed genes (DEGs) in THP-1 cells treated with 100 μM Biochanin A. The most significantly enriched GOBP terms among the downregulated DEGs and total DEGs were associated with DNA replication processes. In contrast, upregulated DEGs were significantly enriched in biological processes related to monocyte differentiation and endoplasmic reticulum (ER) stress. The KEGG pathway enrichment analysis further supported these findings, with downregulated DEGs associated with cell cycle regulation, DNA replication, and steroid biosynthesis pathways (Figure 9d–f). Meanwhile, upregulated DEGs were enriched in KEGG pathways related to metabolic processes and MAPK signaling. The specific gene expression changes involved in the KEGG cell cycle and DNA replication pathways are illustrated in Figure 10.

Therapeutic targeting of spindle assembly checkpoint (SAC) components has emerged as a promising strategy in both preclinical and clinical cancer research [35]. As shown in Figure 10a, key SAC-related genes, including *BUB1*, *BUB3*, *MAD1*, *MAD2*, *BUBR1*, *Aurora B*, and *CDC20*, were significantly downregulated during the G2/M transition in THP-1 cells following Biochanin A treatment, suggesting these genes may represent potential molecular targets. In addition, the majority of genes involved in DNA replication were also downregulated (Figure 10b). These results indicate that Biochanin A disrupts both the G1/S and G2/M phases of the cell cycle, contributing to cell cycle arrest in THP-1 cells.

**Figure 11:**
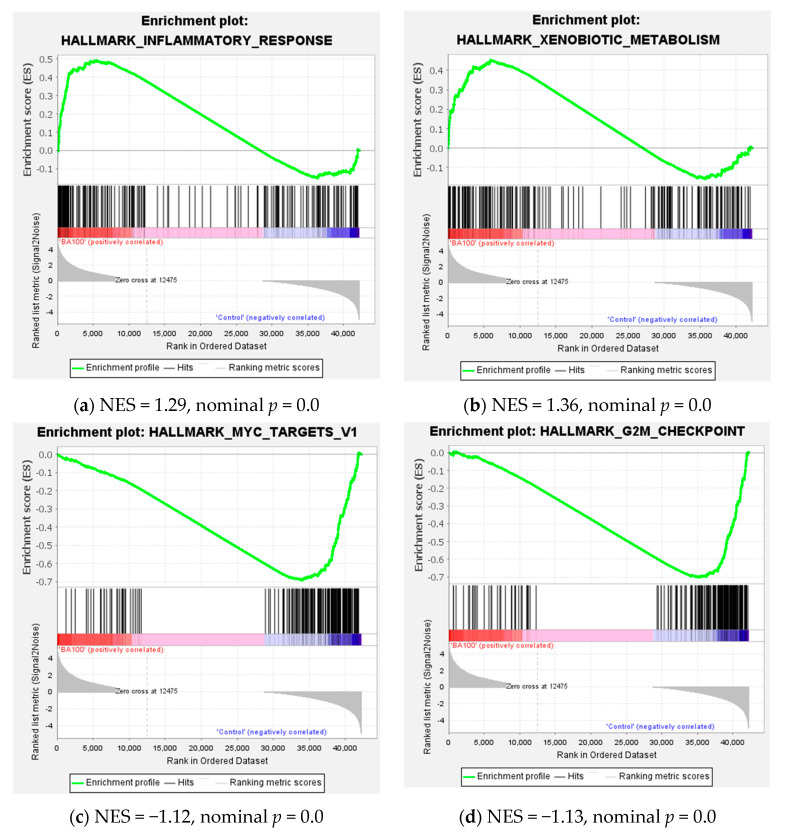
Gene set enrichment analysis (GSEA) plots of Hallmark pathways affected by Biochanin A (100 μM) treatment in THP-1 cells. (**a**,**b**) Gene sets significantly enriched among upregulated genes. (**c**,**d**) Gene sets significantly enriched among downregulated genes.

Consistent with the findings in U937 cells, the gene set enrichment analysis (GSEA) revealed that treatment of THP-1 cells with 100 μM Biochanin A significantly enriched hallmark pathways related to inflammatory response and xenobiotic metabolism among upregulated genes. Conversely, Hallmark pathways associated with MYC targets and G2/M checkpoint were significantly enriched among downregulated genes (Figure 11).

The GOBPs and KEGG pathways enriched with DEGs in response to 200 μM Biochanin A treatment in THP-1 cells are presented in Supplemental Figure S3. Consistent with the findings for 100 μM Biochanin A, the most significantly enriched GOBP terms for downregulated DEGs were related to DNA replication processes, while ER stress pathways were significantly enriched with upregulated DEGs.

The KEGG pathway analysis revealed that cell cycle and DNA replication pathways were enriched with downregulated DEGs, consistent with the findings for 100 μM Biochanin A. In contrast, metabolism and MAPK signaling pathways were enriched with upregulated DEGs (Figure S3). A detailed analysis of the apoptosis pathway (Figure S4) showed that treatment with 200 μM Biochanin A activated both extrinsic apoptosis and TNF-α-mediated apoptosis. Additionally, ER stress-related genes such as *PERK, CHOP, NOXA,* and *PUMA* were upregulated alongside caspases *CASP6, CASP7, CASP9*, and *CASP10*, which are critical mediators of apoptosis.

CHOP (C/EBP homologous protein), also known as GADD153, is a transcription factor that plays a pivotal role in the unfolded protein response (UPR)-mediated apoptotic process [36]. CHOP functions as a key regulator in determining the role of ER stress on AML fate, shifting it from an adaptive to a terminal phase in response to treatments [37]. In our study, we observed a significant induction of CHOP protein expression following treatment with Biochanin A at concentrations of 100 μM and 200 μM. Notably, CHOP protein expression was upregulated at 24 h in U937 cells, whereas in THP-1 cells, upregulation was not observed until 38 h (Figure S5). These findings reflect cell-line-specific differences in the timing of ER stress responses. Despite the variation in CHOP expression and Caspase 7 activation, we observed PARP-1 cleavage and cell death as early as 24 h in both U937 and THP-1 cells (Figure 3). This suggests that Biochanin A-induced apoptosis may proceed through additional pathways beyond CHOP-mediated ER stress and Caspase 7 activation, especially at early time points.

Taken together with the data in Figure S6 and Figure 11, these results indicate that both 100 μM and 200 μM Biochanin A treatments similarly enriched Hallmark pathways. Specifically, they upregulated genes involved in inflammatory response and xenobiotic metabolism, while downregulating genes associated with MYC targets and the G2/M checkpoint".

### 2.5. Effects of Biochanin A on AML-Related Gene Expression

**Figure 12:**
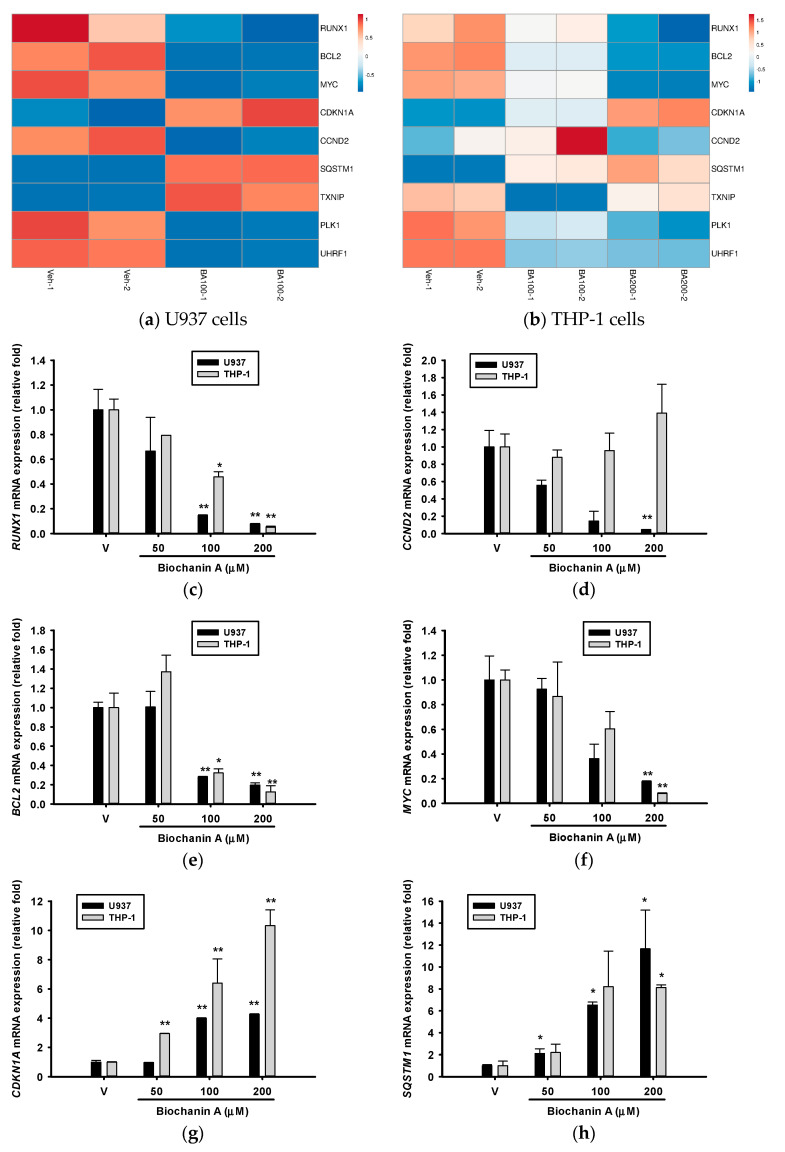

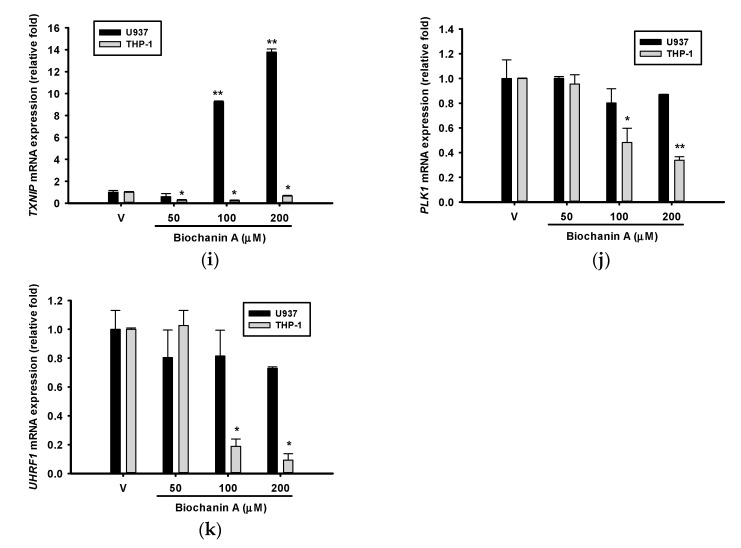
Effects of Biochanin A on the expression of AML-related genes in U937 and THP-1 cells.(**a**,**b**) Heatmaps showing RNA-seq-based expression profiles of the selected AML-related genes following treatment with 100 μM (BA100) and 200 μM (BA200) Biochanin A. (**c**–**k**) mRNA expression levels of individual AML-associated genes were quantified by RT-qPCR 24 h post-treatment, as described in the Materials and Methods section. Data are presented as the mean ± SD of three independent experiments. * *p* < 0.05, ** *p* < 0.01 indicate statistically significant differences compared with vehicle-treated controls.

In our previous studies, we demonstrated that isoflavonoid 8-hydroxydaidzein induced apoptosis by downregulating acute myeloid leukemia (AML)-associated genes such as *RUNX1*, *CCND2*, and *MYC* in U937 cells while upregulating *CDKN1A* (*p21*) and suppressing *BCL2* expression in K562 cells [29,30,31]. In the present study, we examined the expression of these genes as well as those related to autophagy, reactive oxygen species (ROS) production, mitotic regulation, and epigenetic control using RT-qPCR. The corresponding RNA-seq results are shown in Figure 12a,b. Treatment with Biochanin A resulted in the downregulation of *RUNX1*, *BCL2*, *MYC*, *PLK1*, and *UHRF1* and upregulation of *CDKN1A* and *SQSTM1* in both U937 and THP-1 cells. In contrast, the expression patterns of *CCND2* and *TXNIP* differed between the two cell lines.

*RUNX1* is an oncogenic transcription factor and a recognized driver mutation in acute myeloid leukemia (AML), where it plays a key role in regulating *CCND2* expression [38,39]. Both somatic and germline mutations in *RUNX1* are present in approximately 10% of AML cases [40] and are associated with poor clinical prognosis [41]. These mutations contribute to leukemogenesis by enhancing self-renewal capacity and impairing granulocytic differentiation [42]. Targeted therapies against *RUNX1* alterations are currently under investigation for AML treatment [43,44]. As shown in Figure 12c, treatment with Biochanin A (100 μM) significantly reduced *RUNX1* expression by approximately 85% in U937 cells (*p* < 0.01) and 55% in THP-1 cells (*p* < 0.05).

Cyclin D2 (*CCND2*) is a key regulator of the G1/S transition and plays an important role in cell cycle progression and the pathogenesis of acute myeloid leukemia (AML). The overexpression of *CCND2* in AML cells is associated with uncontrolled proliferation and impaired differentiation [45]. As shown in Figure 12d, treatment with Biochanin A resulted in a dose-dependent downregulation of *CCND2* mRNA expression in U937 cells, with a statistically significant reduction observed at 200 μM (*p* < 0.05). These results suggest that Biochanin A may induce apoptosis in U937 cells, at least in part, by targeting the *RUNX1/CCND2* regulatory axis. In contrast, no significant changes in *CCND2* expression were detected in THP-1 cells, consistent with the RNA-seq findings shown in Figure 12b. This indicates that *CCND2* may not be a major mediator of Biochanin A’s anti-proliferative effects in THP-1 cells.

The B-cell lymphoma 2 (BCL-2) family of proteins plays a critical role in intrinsic apoptosis. The overexpression of BCL-2 proteins can inhibit apoptosis, leading to resistance to chemotherapy in AML. AML has been considered a “graveyard” for drug development programs. However, a breakthrough occurred in 2017 with the emergence of several new drugs, including venetoclax, a selective BCL-2 inhibitor, which appears to have reshaped the therapeutic landscape of AML [46]. As shown in Figure 12e, treatment with 100 μM Biochanin A reduced *BCL-2* mRNA expression by approximately 30% in both U937 and THP-1 cells (*p* < 0.01, 0.05), with the downregulation effect showing a dose-dependent relationship.

c-Myc, a transcription factor, regulates the expression of approximately 15% of genes in the genome and plays a crucial role in cell proliferation [26,27,47]. Among its diverse functions, the regulation of ribosome biogenesis is considered its most fundamental and evolutionarily conserved role [48]. A previous GSEA demonstrated that gene sets categorized under ‘Hallmark MYC Targets’ were predominantly downregulated by Biochanin A in U937 and THP-1 cells (Figure 7d and Figure 11c). The RT-qPCR analysis confirmed that treatment with 200 μM Biochanin A led to a significant 90% reduction in *MYC* mRNA expression (*p* < 0.01; Figure 12f). Furthermore, the Western blot analysis revealed that Biochanin A inhibited c-Myc protein expression in U937 and THP-1 cells (Figure 3), underscoring c-Myc as a central molecular target in this context.

c-Myc directly induces the expression of AP4, which in turn represses *CDKN1A* (*p21*) transcription, a cyclin-dependent kinase (CDK) inhibitor [49]. *p21* is a critical regulator of the cell cycle, DNA damage response, and apoptosis. Its expression is controlled through both p53-dependent and p53-independent pathways, leading to G1 phase arrest and apoptosis [50]. Inducing *CDKN1A* (*p21*) expression to promote cell cycle arrest and trigger apoptosis has been proposed as a potential therapeutic strategy for AML [51]. Figure 12g demonstrates that Biochanin A (50–200 μM) upregulated *CDKN1A* mRNA expression in a dose-dependent manner in both U937 and THP-1 cells, with a more pronounced effect observed in THP-1 cells. Additionally, as shown in Figure 10a, the KEGG cell cycle pathway map revealed the downregulation of CDKs upon Biochanin A treatment in THP-1 cells. Collectively, these findings suggest that the *MYC*/*CDKN1A* axis plays a pivotal role in controlling cell proliferation and inducing cell cycle arrest in response to Biochanin A treatment.

p62/SQSTM1 is a multifunctional scaffold protein involved in autophagy, apoptosis, and multiple cellular signaling pathways [52]. Previous studies have shown that both mRNA and protein levels of *p62/SQSTM1* are significantly upregulated during the differentiation of AML cells into neutrophils and granulocytes [52]. In the present study, treatment with Biochanin A (50–200 μM) led to a dose-dependent increase in *p62/SQSTM1* mRNA expression in U937 cells, suggesting the induction of autophagy or differentiation. A similar upregulation was observed in THP-1 cells treated with 200 μM Biochanin A (Figure 12h).

Thioredoxin-interacting protein (*TXNIP*) is a critical regulator of oxidative stress and cellular metabolism. Previous studies have demonstrated that the combination of chemotherapeutic agents with differentiation inducers enhances *TXNIP* expression and improves therapeutic efficacy in AML cells [53]. Similarly, treatment with the histone methyltransferase inhibitor DZNep was shown to upregulate *TXNIP*, leading to increased reactive oxygen species (ROS) production and subsequent cell death in AML cell lines [54]. In the present study, treatment with Biochanin A (100–200 μM) significantly increased *TXNIP* mRNA expression—by over 10-fold—in U937 cells, while a dose-dependent decrease in *TXNIP* expression was observed in THP-1 cells (Figure 12i). This differential regulation may partially explain the greater cytotoxic effects observed in U937 cells compared to THP-1 cells (Figure 2).

Polo-like kinase 1 (*PLK1*) is a serine/threonine-protein kinase that serves as a central regulator of mitosis, particularly at the G2/M transition [55]. It is frequently overexpressed in various malignancies, including AML [55,56], and several *PLK1* inhibitors are under development as anticancer therapeutics [57,58,59]. In our study, Biochanin A treatment reduced *PLK1* mRNA expression in THP-1 cells, as detected by RT-qPCR (Figure 12j), but not in U937 cells—contrasting with RNA-seq results shown in Figure 12a. The KEGG pathway analysis (Figure 10a) revealed that downregulated genes in THP-1 cells were enriched in the G2/M checkpoint and transition pathways. These findings suggest that Biochanin A-induced cytotoxicity in THP-1 cells may involve cell cycle arrest at the G2/M phase.

Ubiquitin-like with PHD and RING Finger domains 1 (*UHRF1*) is an epigenetic regulator involved in DNA methylation and histone modification. It is highly expressed in AML and associated with poor clinical outcomes [60]. The suppression of *UHRF1* downregulates leukemia stem cell signatures and *MYC*-related pathways in AML cells [60]. Moreover, *UHRF1* negatively regulates THP-1 cell differentiation, and its depletion sensitizes cells to phorbol 12-myristate 13-acetate (PMA) while promoting pro-inflammatory cytokine expression [61]. Several small-molecule *UHRF1* inhibitors have been developed [62,63,64]. In the present study, Biochanin A treatment (100 and 200 μM) significantly reduced *UHRF1* mRNA expression in THP-1 cells to 19% and 9% of vehicle-treated controls, respectively (Figure 12k). In contrast, no significant reduction was observed in U937 cells, differing from the RNA-seq findings presented in Figure 12a. These results suggest that Biochanin A may mimic PMA-like effects, leading to G2/M phase arrest and promoting differentiation in THP-1 cells [65].

The *MYB* gene family consists of three members—*MYB*, *MYBL1*, and *MYBL2*—which encode transcription factors predominantly expressed in hematopoietic tissues. *MYB* functions upstream of *MYC* and *BCL2* and has been shown to transcriptionally regulate their expression, contributing to leukemogenic programs in AML [66,67]. Additionally, research indicates that *RUNX1* is essential for maintaining enhancer activity associated with both *MYB* and *MYC*, highlighting the interplay among these genes in leukemogenesis [68].

RNA-seq data examining the effects of Biochanin A on *MYB*, *MYBL1*, and *MYBL2* expression in U937 and THP-1 cells are shown in Figure S7. In U937 cells, treatment with Biochanin A (100 μM) downregulated *MYB* and *MYBL2* expression by 37% and 21%, respectively (Figure S7a). In THP-1 cells, *MYBL2* expression was significantly and dose-dependently reduced by Biochanin A (100 and 200 μM). In contrast, *MYB* was inhibited only at 100 μM, while *MYBL1* showed inhibition only at 200 μM (Figure S7b). Notably, *MYBL2* overexpression is a known prognostic factor in AML, identifying a subset of patients with poor outcomes [69]. These findings suggest that Biochanin A may downregulate members of the *MYB* gene family, although further validation by RT-qPCR is needed.

## 3. Discussion

### 3.1. Transcriptomic Effects of Biochanin A in U937 and THP-1 Cells

This study aims to elucidate the molecular responses of AML cell lines, U937 and THP-1, to Biochanin A through genome-wide gene expression profiling. To interpret the transcriptomic changes, we employed two widely adopted enrichment strategies: (i) over-representation analysis (ORA), which evaluates whether significantly differentially expressed genes are enriched within predefined gene sets, and (ii) gene set enrichment analysis (GSEA), which examines whether members of a gene set are non-randomly distributed near the top or bottom of a ranked list of all genes based on the magnitude and direction of expression change. The gene sets analyzed encompass biological processes from Gene Ontology (GO) [70], canonical pathways from KEGG [71], and curated experimental gene sets from the Molecular Signatures Database (MSigDB) [72].

An ORA depends on predefined significance thresholds for selecting differentially expressed genes, which may result in the exclusion of gene sets exhibiting modest but coordinated changes. In contrast, functional class scoring (FCS) methods such as GSEA utilize the entire expression dataset without applying arbitrary cutoffs, thereby enhancing sensitivity to subtle transcriptional shifts and improving reproducibility across independent experiments [22,73].

In U937 cells treated with 100 μM Biochanin A, upregulated DEGs were significantly enriched in GOBP terms related to innate immune and inflammatory responses. Conversely, downregulated DEGs were primarily involved in RNA processing and cholesterol biosynthesis. The KEGG pathway analysis revealed significant enrichment of TNF signaling and amino acid metabolism among upregulated genes, while steroid biosynthesis and RNA polymerase pathways were suppressed.

In THP-1 cells treated with 100 and 200 μM Biochanin A, downregulated DEGs were significantly enriched in GOBPs and KEGG pathways associated with DNA replication and cell cycle regulation. In contrast, upregulated DEGs were enriched in endoplasmic reticulum (ER) stress-related pathways.

The GSEA further confirmed the positive enrichment of Hallmark gene sets related to inflammatory response, xenobiotic metabolism, and apoptosis as well as the negative enrichment of MYC targets, G2/M checkpoint, and cholesterol homeostasis in both cell lines. Notably, the Western blot analysis (Figure 3) revealed reduced c-Myc protein expression in both U937 and THP-1 cells following Biochanin A treatment, supporting the transcriptomic findings of MYC target downregulation. Given that c-Myc is a key regulator of cell proliferation and biosynthetic activity in acute myeloid leukemia (AML), its suppression likely contributes to the inhibition of cell cycle progression, cholesterol biosynthesis, and pro-survival signaling. Concurrently, the induction of ER stress and inflammatory pathways suggests the activation of pro-apoptotic or adaptive stress responses.

Collectively, these results highlight that Biochanin A exerts anti-leukemic effects through multi-targeted transcriptional reprogramming, including the downregulation of MYC-driven proliferative networks and activation of immune and stress-related pathways. These convergent mechanisms across both AML cell lines support the therapeutic potential of Biochanin A in leukemia treatment.

### 3.2. Possible Cell-Type-Specific Mechanisms of Action of Biochanin A

Biochanin A treatment in U937 cells upregulated *TXNIP*, a key oxidative stress regulator that inhibits thioredoxin and sensitizes cells to reactive oxygen species (ROS)-induced apoptosis [74]. Concurrently, *CCND2*, a G1 cyclin involved in cell cycle progression, was downregulated, indicating a potential G1 phase arrest. The coordinated induction of oxidative stress via *TXNIP* and inhibition of proliferative signaling via *CCND2* downregulation implies that Biochanin A promotes apoptosis and suppresses proliferation in U937 cells through redox imbalance and cell cycle blockade.

In THP-1 cells, Biochanin A downregulated *PLK1* and *UHRF1*, genes essential for mitotic progression and epigenetic maintenance, respectively. PLK1 is a serine/threonine kinase that governs centrosome maturation, spindle assembly, and G2/M transition, and its suppression is associated with cell cycle arrest and mitotic catastrophe in cancer cells [75]. Similarly, UHRF1 plays a central role in DNA methylation and chromatin remodeling, and its downregulation may impair epigenetic stability and promote apoptosis [76]. These transcriptional changes are in agreement with the ORA, which revealed a significant downregulation of the KEGG cell cycle pathway following Biochanin A treatment in THP-1 cells. Together, these results suggest that Biochanin A disrupts cell cycle progression and chromatin homeostasis, thereby contributing to reduced proliferation and increased cell death.

### 3.3. Potential Off-Target Effects of Biochanin A Based on Transcriptomic Changes

While Biochanin A demonstrated transcriptomic signatures consistent with anti-leukemic activity, including the suppression of MYC targets and cell cycle-related genes, several differentially expressed genes (DEGs) and enriched pathways may reflect potential off-target or pleiotropic effects that warrant further consideration.

First, in both U937 and THP-1 cells, Biochanin A treatment led to the upregulation of genes associated with inflammatory and immune responses, including TNF signaling and interferon pathways. Although such responses may contribute to anti-tumor immunity, recent studies have shown that activation of the innate immune pathway can paradoxically promote tumor progression [77].

Second, alterations in rRNA processing and splicesome among downregulated DEGs raise the possibility that Biochanin A may affect global gene expression or protein synthesis machinery beyond its primary targets. Although this may contribute to its anti-proliferative effects, such broad interference could impair normal cellular functions in rapidly dividing non-cancerous cells, such as gastrointestinal epithelium [78].

Finally, the downregulation of cholesterol and steroid biosynthesis pathways, while detrimental to leukemic cell survival, may also affect normal lipid homeostasis. If replicated in vivo, such effects could potentially impact membrane fluidity, hormone synthesis, or lipid-mediated signaling in non-malignant tissues [79].

### 3.4. Potential Usage of Biochanin A in TP53-Mutant AML Cells

Consistent with the behavior of most phytoestrogens [29,80,81,82], Biochanin A exhibits multi-target potential against AML, including inhibiting cell proliferation, the induction of cell cycle arrest, and the promotion of apoptosis. These effects appear to be primarily mediated through the central regulation of the c-Myc/p21 axis, highlighting its pivotal role in the activity of Biochanin A against AML. Notably, these effects were observed in U937 and THP-1 cells, both of which harbor *TP53* mutations [18].

The *TP53* gene encodes the tumor suppressor protein p53, which is activated in response to DNA damage and mediates cell cycle arrest, DNA repair, senescence, or apoptosis. Although *TP53* mutations are found in approximately 10% of AML patients [83], these cases are associated with particularly poor prognosis, resistance to standard chemotherapy, and high relapse rates [84,85,86]. Given that most conventional chemotherapies rely on functional p53 to induce apoptosis, treatment strategies for *TP53*-mutant AML must focus on p53-independent mechanisms [84].

In this study, we report that Biochanin A downregulates oncogenes *RUNX1, BCL2*, and *c-MYC*, which are key regulators of leukemogenesis. In addition, Biochanin A also upregulates *CHOP (GADD153)*, *CDKN1A (p21)*, and *SQSTM1 (p62)*. Although *p21* is a well-known transcriptional target of p53, it can also be upregulated independently of p53 via alternative transcription factors such as CHOP, particularly under conditions of cellular stress [87]. Conversely, *p21* expression can be repressed by AP4, a transcriptional repressor that is directly induced by c-Myc [49]. These findings support the notion that Biochanin A exerts its anti-leukemic effects through p53-independent mechanisms, offering a promising therapeutic approach for targeting *TP53*-mutant AML. Further in vivo studies and clinical investigations are warranted to validate its efficacy, either as a standalone treatment or in combination with other therapeutic regimens.

### 3.5. Limitations of the Currrent Study

One limitation of this study is the lack of direct apoptosis and cell cycle analyses, which would have provided deeper mechanistic insight into the anti-leukemic effects of Biochanin A. Due to current constraints, we focused on cell viability assays, Western blotting, and transcriptomic profiling. Future investigations incorporating flow cytometry or related assays are warranted to validate and expand upon these findings.

Despite exhibiting favorable drug-likeness and predicted pharmacokinetic properties, the clinical application of Biochanin A remains limited due to its poor aqueous solubility and rapid systemic clearance, which render the 100–200 μM concentrations used in this study difficult to achieve in vivo [1,3,7]. To address these limitations and enhance therapeutic efficacy, several advanced drug delivery strategies have been explored. These include solid dispersion techniques [88], nanostructured lipid carriers (NLCs) [89], PEGylated nanostructured lipid carriers (PEG-NLC) [90], self-assembled micelle systems [91], and nano-sized phospholipid complexes [92].

Recent studies employing Biochanin A-loaded nanoparticles have shown improved pharmacokinetics, including prolonged circulation time and enhanced tissue accumulation, particularly in tumor-bearing animal models. For instance, BCA-NLC showed higher AUC value and circulated in blood for a longer time than BCA suspension. The studies demonstrated that NLC could be a potential delivery system for BCA to improve bioavailability [89]. PEG-NLC formulations have been shown to significantly increase the plasma half-life and bioavailability of flavonoids, including isoflavones such as Biochanin A, compared to conventional formulations [90]. Such systems have demonstrated enhanced solubility, stability, and sustained release profiles in preclinical models.

## 4. Materials and Methods

### 4.1. Chemicals

Biochanin A was purchased from Cayman Chemical (Ann Arbor, MI, USA). The other chemicals were from Sigma-Aldrich Co. (St. Louis, MO, USA) unless otherwise specified.

### 4.2. Cell Culture

U937 and THP-1 cell lines were obtained from the Bio-resource Collection and Research Center (Hsinchu, Taiwan). U937 cells were cultured in RPMI-1640 Hybri-Max medium supplemented with 10% fetal bovine serum (FBS), 1% nonessential amino acids (NEAA), 100 U/mL penicillin, and 100 µg/mL streptomycin (Thermo Fisher Scientific, Rockford, IL, USA). For THP-1 cells, the same medium was used with the addition of 1 mM sodium pyruvate and 0.05 mM 2-mercaptoethanol (Thermo Fisher Scientific). Cells were maintained in a 5% CO_2_ incubator at 37 °C and passaged twice weekly to maintain a density of 2 × 10^5^ to 10^6^/mL.

### 4.3. Cell Proliferation and Viability Analysis

U937 and THP-1 cell lines (5 × 10^5^ cells/mL) were treated with either vehicle control (0.1% DMSO) or varying concentrations of Biochanin A (12.5–200 µM) for 24 h to assess cell proliferation. Following treatment, cell proliferation was measured using the MTT assay. Briefly, 1/10 volume of 0.5% MTT solution (3-(4,5-dimethylthiazol-2-yl)-2,5-diphenyltetrazolium bromide in PBS) was added to each well, and cells were incubated for an additional 3 h. Subsequently, the MTT-containing medium was removed by centrifugation, and the resulting formazan crystals were solubilized in DMSO. Absorbance was then measured at 550 nm using a spectrophotometer, as previously described [93].

To evaluate cytotoxicity, U937 and THP-1 cells were treated with Biochanin A (50–200 µM) or the positive control Ara-C (cytarabine; 0.25 and 0.5 µM). Cell viability was assessed using the trypan blue exclusion assay. Following treatment, cells were mixed with trypan blue dye and immediately counted using a hemocytometer in accordance with established protocols for suspension cultures [94].

### 4.4. Western Blot Analysis

Following treatment with Biochanin A for the indicated period, U937 and THP-1 cells were lysed using RIPA lysis buffer to prepare cell lysates (Cayman, Ann Arbor, MI, USA). The protein concentration was quantified by the Bradford binding assay (Bio-Rad Laboratories, Hercules, CA, USA) against bovine serum albumin (BSA) standard.

Equal amounts of total protein were separated by SDS-PAGE (8–10%) and transferred onto polyvinylidene difluoride (PVDF) membranes (HyBond-P, GE Healthcare, Chicago, IL, USA) using CAPS transfer buffer (10 mM, pH 10.5, containing 10% methanol) at 20 V overnight at 4 °C. Following transfer, membranes were blocked for 2 h at room temperature in PBS containing 0.05% Tween-20 (PBST, pH 7.4) supplemented with 5% skim milk. Membranes were subsequently sectioned based on the molecular weights of the target proteins and incubated overnight at 4 °C with the respective primary antibodies (see Table 1). After washing, membranes were incubated with horseradish peroxidase (HRP)-conjugated secondary antibodies (Jackson ImmunoResearch, West Grove, PA, USA) for 1 h at room temperature. Protein bands were visualized using an enhanced chemiluminescence detection system (GE Healthcare), and band intensities were quantified using ImageJ software (version 1.54p) [95].

For detection of Caspase 7 and cleaved Caspase 7, we used two different antibodies: a total Caspase 7 antibody (capable of recognizing both full-length and cleaved forms when present at sufficient levels) and a cleaved Caspase 7-specific antibody. In some experiments (e.g., THP-1 cells), where the cleaved form was less abundant, the membrane was first probed with the total Caspase 7 antibody. After imaging, the same membrane was subsequently re-probed with the cleaved Caspase 7 antibody without stripping. This approach allowed us to visualize both total and cleaved forms from the same blot, improving detection sensitivity while preserving protein integrity. In other case (e.g., U937 cells), the cleaved form was detectable using the total Caspase 7 antibody alone, and a single blot was used.

### 4.5. Total RNA Isolation and RNA Sequencing

Total RNA was isolated with the Illustra RNA Spin Mini RNA Isolation Kit (GE Healthcare). RNA samples were sent to Taiwan Genomic Industry Alliance Inc. (Taipei, Taiwan) for RNA sequencing using Illumina NovaSeq X Plus. HISAT2 aligner was used for mapping NGS reads against human reference genome version GRCh38, and FastQC Version 0.11.9 was used for quality control check. DESeq2 was used for analyzing differential analysis of count data [96].

### 4.6. Overrepresentation Analysis (ORA) and Gene Set Enrichment Analysis (GSEA)

To investigate the biological functions and significantly enriched pathways associated with the differentially expressed genes (DEGs), Gene Ontology (GO) term and Kyoto Encyclopedia of Genes and Genomes (KEGG) pathway enrichment analyses were performed using the Database for Annotation, Visualization, and Integrated Discovery (DAVID, version 2021; https://david.ncifcrf.gov/, accessed on 10 June 2024). DEGs were selected based on an absolute log_2_ fold change > 1 and a *p*-value < 0.05 [97,98,99].

To further validate the enrichment of statistically significant molecular pathways in response to Biochanin A treatment, gene set enrichment analysis (GSEA, version 4.3.2; http://www.gsea-msigdb.org/gsea/index.jsp, accessed on 12 June 2024) was conducted [22].

### 4.7. Reverse Transcription Real-Time PCR (RT-qPCR)

The High-Capacity cDNA Archive kit (Thermo Fisher Scientific) was used for reverse transcription of RNA obtained from Section 4.4. The resulting cDNA was utilized for qPCR using a Power SYBR Green PCR Master Mix (Thermo Fisher Scientific) with the appropriate primer pair (Table 2) at 95 °C for 2 min, 40 cycles at 94 °C for 15 s, and 60 °C for 60 s (ABI StepOne Real-Time PCR System). The relative mRNA expression was normalized with GAPDH expression and then calculated by the 2^−ΔΔCT^ method. Specificity verification was performed by melting curve.

### 4.8. Statistical Analysis

All experiments were repeated at least three times, and the values were expressed as the mean ± SD. The results were analyzed using one-way ANOVA with Dunnett’s post hoc test, and a *p* value < 0.05 was considered statistically significant.

## 5. Conclusions

In this study, we demonstrated that Biochanin A exerts significant anticancer effects on AML cell lines U937 and THP-1 through both shared and distinct mechanisms. Biochanin A consistently inhibited cell proliferation and induced apoptosis in both cell lines by activating caspase-7 and promoting PARP1 cleavage. Common molecular targets included the downregulation of *RUNX1*, *BCL2*, and *MYC* oncogenes as well as the upregulation of *CHOP (GADD153), CDKN1A (p21)*, and *SQSTM1 (p62)*, indicating its ability to regulate apoptosis and cell cycle arrest across both cell lines.

The in silico analysis revealed that Biochanin A upregulated hallmark pathways associated with inflammatory responses and apoptosis, while downregulating gene sets related to MYC targets, cholesterol homeostasis, and cell cycle checkpoints in both cell lines. Notably, *TXNIP* expression was specifically upregulated in U937 cells, whereas *PLK1* and *UHRF1* were downregulated in THP-1 cells, suggesting the involvement of cell-type-specific regulatory mechanisms.

In summary, Biochanin A demonstrates shared effects on key oncogenes and apoptosis regulation while inducing cell-type-specific pathways to achieve cytotoxicity and cell cycle arrest. These findings underscore Biochanin A as a promising multi-targeted therapeutic agent for AML, with distinct mechanisms of action depending on cellular context. Further in vivo studies and clinical validations are warranted to fully explore its therapeutic potential.

## Figures and Tables

**Table 1:** Primary antibody used for Western blot.

Antibody	Company	Catalog Number
β-Actin	Proteintech	20536-1-AP
Caspase 7	Cell Signaling	9492
Cleaved Caspase 7	Cell Signaling	9491
BCL-2	Genetex	GTX100064
CHOP/GADD153	Genetex	GTX112827
c-Myc	Genetex	GTX103436
PARP-1	Santa cruz	sc-7150

**Table 2:** The primer pairs used in qPCR.

Gene	Primer Sequence (5′-3′)		Size (bp)
*GAPDH*	F	CATGAGAAGTATGACAACAGCCT	113
	R	AGTCCTTCCACGATACCAAAGT	
*CCND2*	F	TTTGCCATGTACCCACCGTC	104
	R	AGGGCATCACAAGTGAGCG	
*BCL2*	F	GGTGGGGTCATGTGTGTGG	89
	R	GTCGTTTCTTGCCACTGATGA	
*MYC*	F	CGGTTCAGGTACTCAGTCATCC	162
	R	TTGGACGGACAGGATGTATGC	
*CDKN1A*	F	AGGTGGACCTGGAGACTCTCAG	95
	R	TCCTCTTGGAGAAGATCAGCCG	
*SQSTM1 (p62)*	F	AAGCCGGGTGGGAATGTTG	116
	R	CCTGAACAGTTATCCGACTCCAT	
*RUNX1*	F	CTTGTCTCCACTGAGGCACA	133
	R	CTGTGTAGGGGAGCCACATT	
*TXNIP*	F	ATATGGGTGTGTAGACTACTGGG	103
	R	GACATCCACCAGATCCACTACT	
*PLK1*	F	TGACTCAACACGCCTCATCC	155
	R	GCTCGCTCATGTAATTGCGG	
*UHRF1*	F	AGGTGGTCATGCTCAACTACA	116
	R	CACGTTGGCGTAGAGTTCCC	

## Data Availability

The data presented in this study are available in the submission system and as Supplementary Material.

## Supplementary Materials

The following supporting information can be downloaded at https://www.mdpi.com/article/10.3390/ijms26115317/s1.
