# Supplementary material for: Molecular Mechanisms of Biochanin A in AML Cells: Apoptosis Induction and Pathway-Specific Regulation in U937 and THP-1
Source: Int J Mol Sci. 2025 May 31;26(11):5317. doi: 10.3390/ijms26115317 (PMC12154116; doi:10.3390/ijms26115317)
Supplement: Supplementary file 1 [file ijms-26-05317-s001.zip › ijms-3640082-supplementary.pdf]

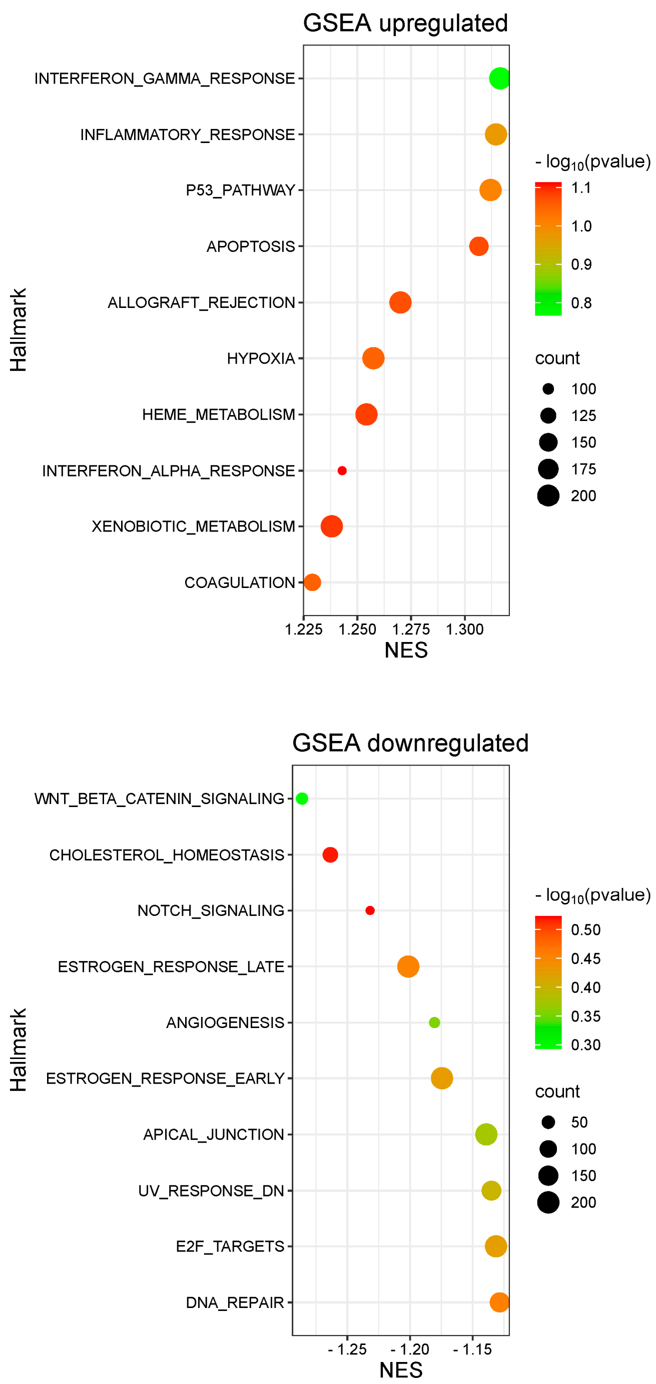

Figure S1. Top 10 hallmark gene sets enriched with upregulated and downregulated gene sets in Biochanin A (100 μM)-treated U937 cells.

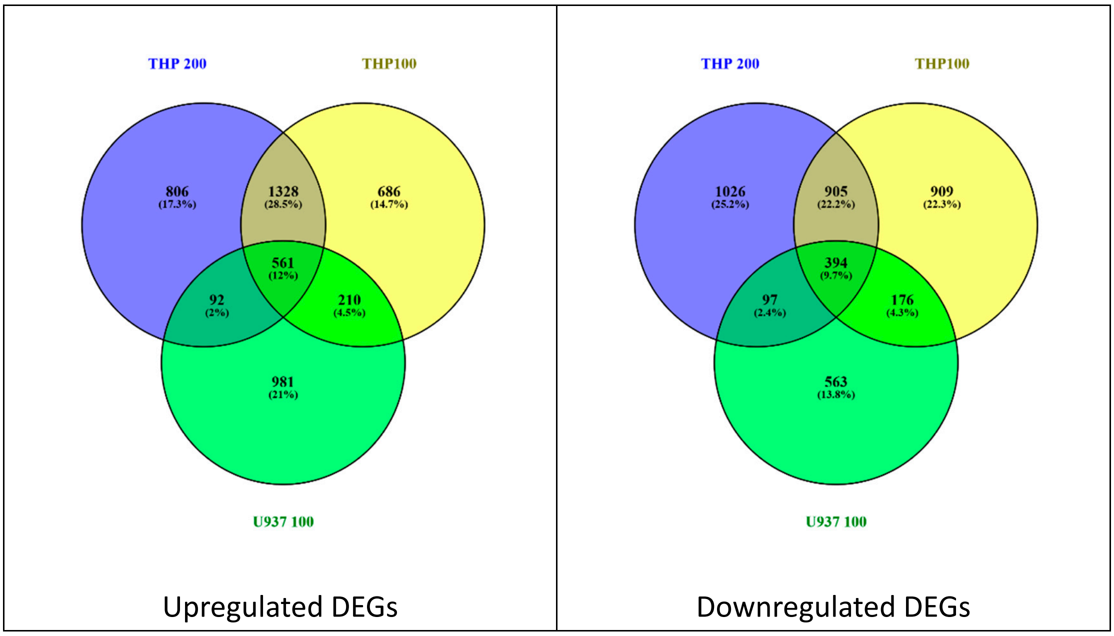

Figure S2. Common DEGs upregulated and downregulated by Biochanin A in THP-1 cells and in U937 cells. There figures were generated by Venny 2.1 on line tool (https://bioinfogp.cnb.csic.es/tools/venny/index.html, access on 2024/12/15)

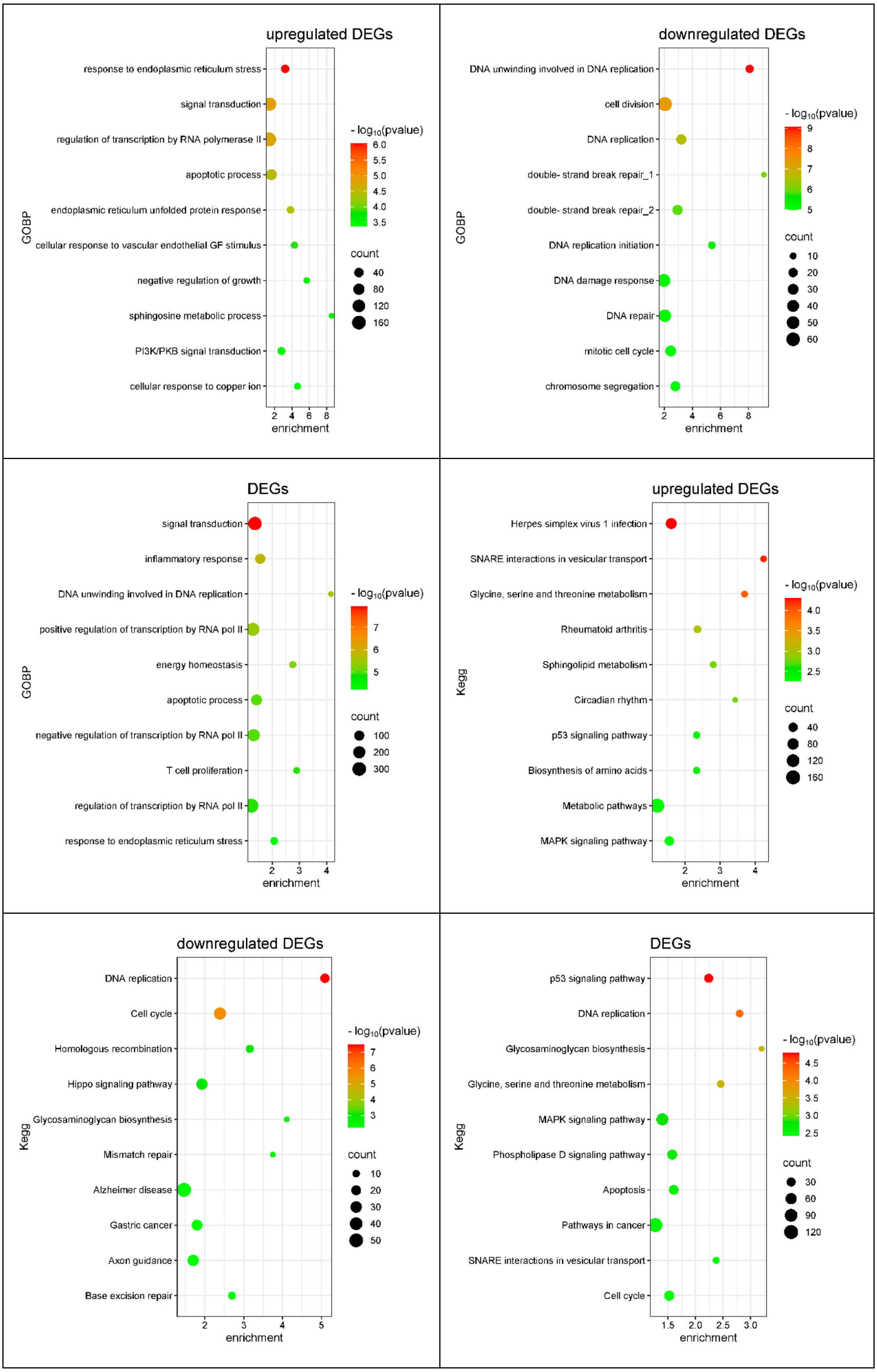

Figure S3. GOBP and KEGG pathways enriched by Biochenin A (200 μM)-caused DEGs in THP-1 cells.

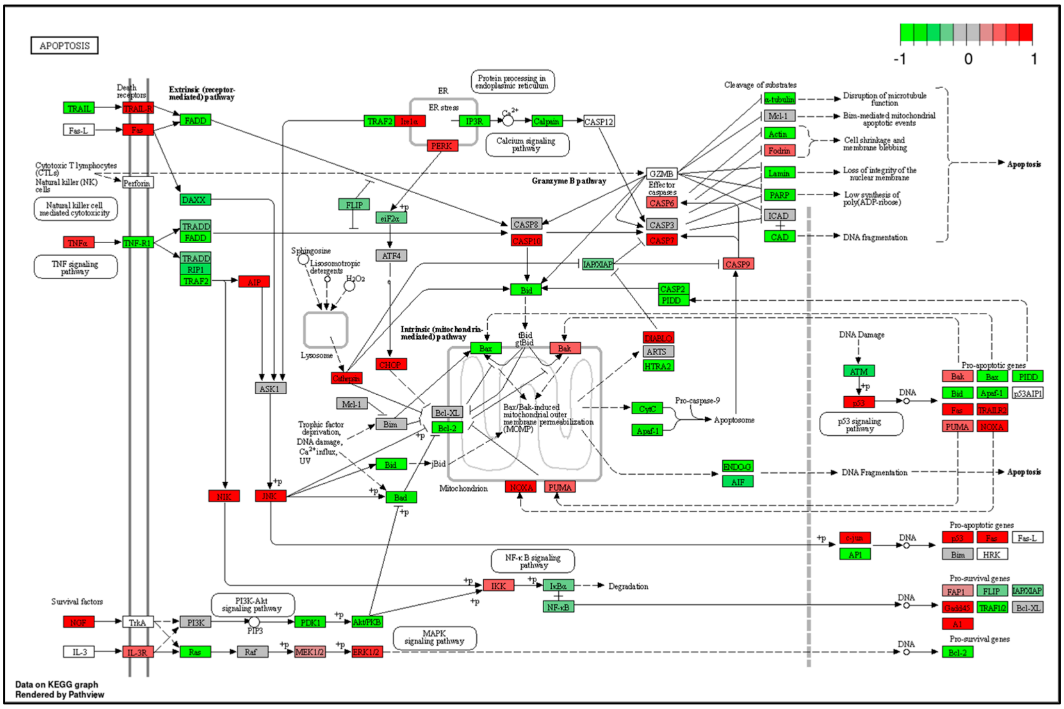

Figure S4. KEGG apoptosis pathway enriched by DEGs caused by Biochenin A (200 μM) in THP-1 cells

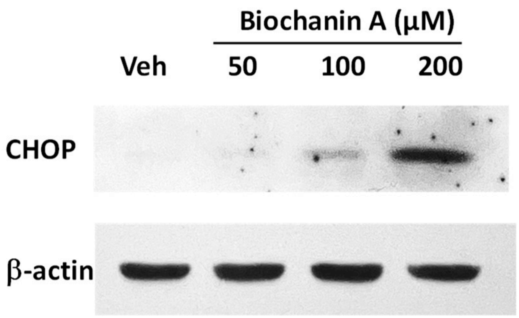

24h
U937 cells
(a)

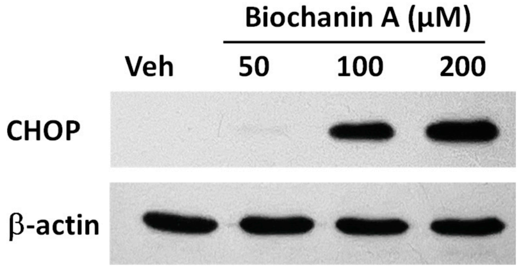

38 h
THP-1 cells
(b)

Figure S5. Effects of Biochanin A on CHOP protein expression in U937 and THP-1 cells. Western blot was performed as described in Materials and Methods.

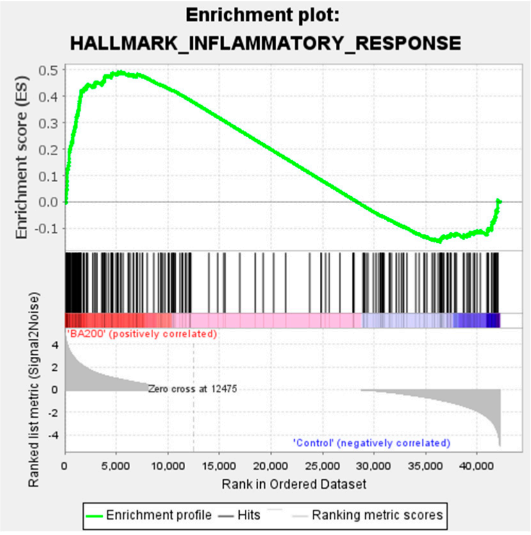

NES=1.28, nominal *p*=0.0

(a)

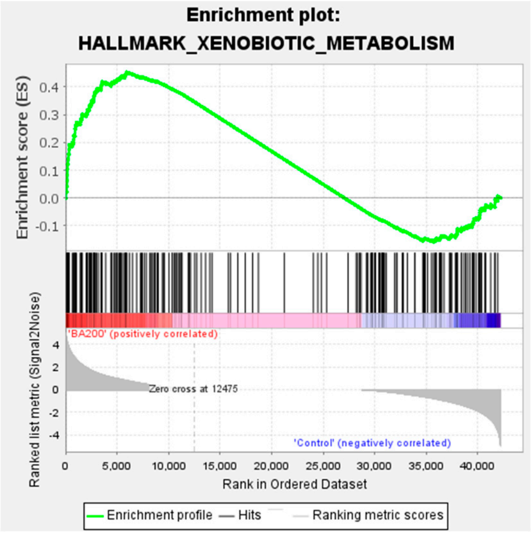

NES=1.36, nominal *p*=0.0

(b)

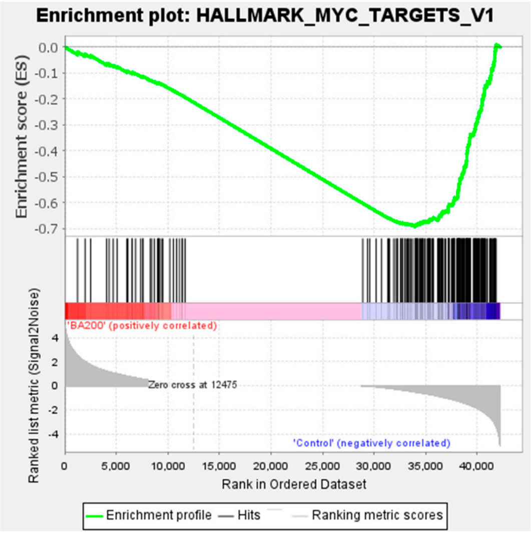

NES = -1.12, nominal *p*=0.0

(c)

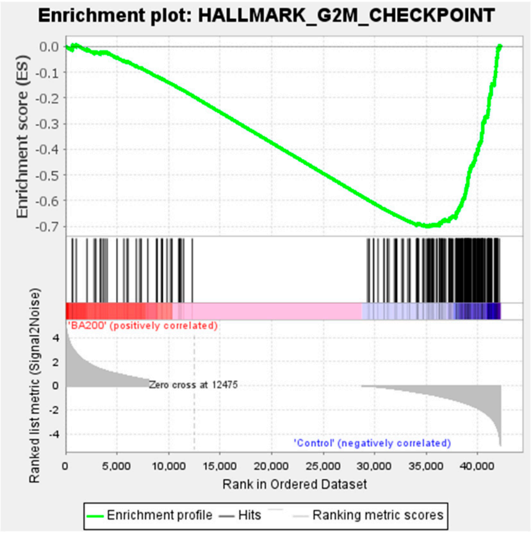

NES= -1.12, nominal *p*=0.0

(d)

Figure S6. GSEA enrichment plots of gene sets affected by Biochanin A (200 μM) treatment in THP-1 cells. (a, b) Gene sets enriched with upregulated genes. (c, d) Gene sets enriched with downregulated genes.

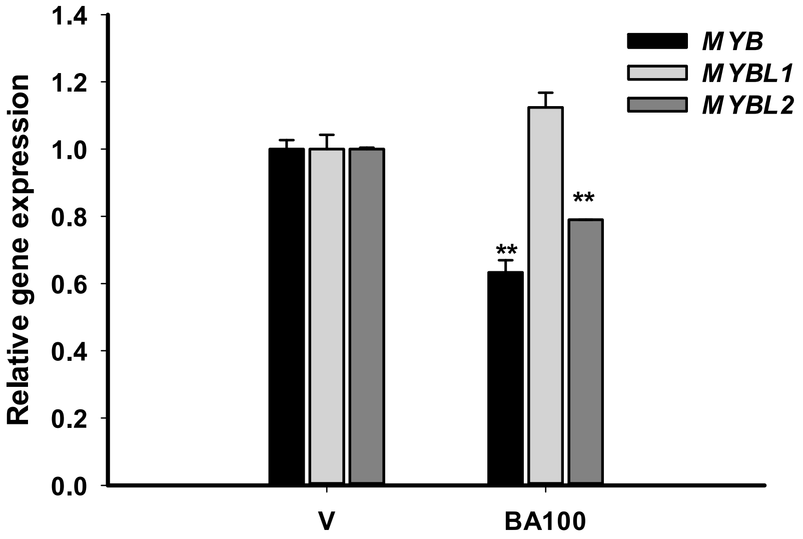

U937 cells

(a)

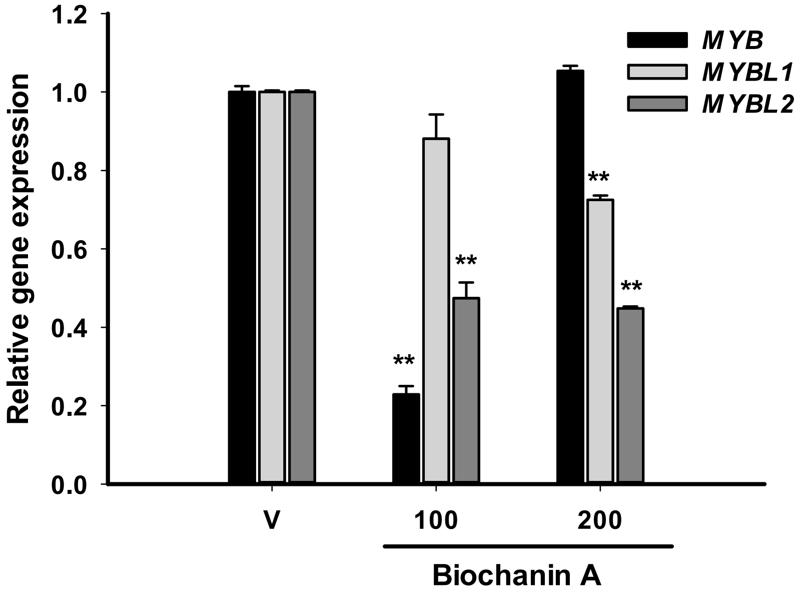

THP-1 cells

(b)

Figure S7. The expression patterns of *MYB* family genes in response to Biochanin A treatment, as determined by RNA-seq analysis, in U937 (a) and THP-1 cells (b).
